# β‐Adrenergic Receptor Activation Modulates the Induction of Complex Spike‐Dependent LTP by Regulating Multiple Forms of Heterosynaptic Plasticity

**DOI:** 10.1002/hipo.70043

**Published:** 2025-10-15

**Authors:** Thomas J. O'Dell

**Affiliations:** ^1^ Department of Physiology, David Geffen School of Medicine, and Integrative Center for Learning and Memory, Brain Research Institute University of California Los Angeles California USA

**Keywords:** β‐adrenergic receptor, BTSP, complex spike burst, heterosynaptic plasticity, LTP, synaptic competition, synaptic cooperativity

## Abstract

Norepinephrine, acting through β‐adrenergic receptors (β‐ARs), has a key role in hippocampus‐dependent forms of learning. Although β‐AR activation also facilitates the induction of Hebbian LTP, recent findings indicate that a non‐Hebbian form of synaptic plasticity, known as behavioral timescale synaptic plasticity (BTSP), underlies hippocampus‐dependent spatial learning. To explore the role of noradrenergic signaling in BTSP, I investigated the effects of the β‐AR activation on complex spike (CS) burst‐dependent LTP, a form of BTSP induced by theta‐pulse stimulation (TPS) protocols in the CA1 region of mouse hippocampal slices. β‐AR activation not only enhanced the homosynaptic potentiation of synaptic transmission induced by TPS but also modulated heterosynaptic forms of plasticity critical for CS burst‐dependent LTP induction. Specifically, β‐AR activation enhanced the heterosynaptic facilitation of CS bursting induced by brief TPS trains and facilitated the ability of synapses to interact in a cooperative fashion to undergo LTP, even when independent groups of synapses were activated up to 10 s apart. β‐AR activation also enhanced a CS burst‐dependent form of heterosynaptic depression elicited by longer trains of TPS, resulting in a winner‐take‐all form of synaptic competition where the β‐AR‐mediated facilitation of LTP induction at one group of synapses was associated with a strong, heterosynaptic suppression of LTP at other synapses. Together, these findings indicate that β‐AR activation dynamically regulates fundamental properties of CS burst‐dependent synaptic plasticity by modulating multiple forms of heterosynaptic plasticity.

## 1 Introduction

The modulatory neurotransmitter norepinephrine (NE), acting through β‐adrenergic receptors (β‐ARs), enhances learning and memory (Sara 2009; Hagena et al. 2016) as well as the induction of Hebbian LTP, a form of synaptic plasticity thought to underlie memory formation (O'Dell et al. 2015). Importantly, associative forms of learning are highly supervised and dependent on factors such as continency (i.e., the predictive value of a conditional stimulus), attention, and prediction errors (Gallistel and Matzel 2013; Fanselow and Wassum 2016). In contrast, Hebbian LTP is a correlation‐based form of plasticity where increases in synaptic strength can be induced in an unsupervised manner by coincident pre‐ and postsynaptic activity. Thus, although considerable evidence supports the notion that Hebbian LTP is involved in memory formation (Dringenberg 2020), the unsupervised nature of changes in synaptic strength produced by Hebbian LTP is strikingly incompatible with fundamental properties of associative learning (Gallistel and Matzel 2013). Notably, noradrenergic neurons in the locus coeruleus (LC) are tonically active during heightened levels of arousal and attention (Berridge and Waterhouse 2003). In addition, LC neurons are also activated by novel stimuli (Sara et al. 1994; Takeuchi et al. 2016), changes in stimulus–reward contingencies (Sara and Segal 1991; Bouret and Sara 2004), and prediction errors (Jorden 2024). These findings indicate that LC neurons encode information critical for associative memory formation. Thus, the noradrenergic modulation of Hebbian LTP may provide a mechanism that allows synapses to use a simple, correlation‐based form of plasticity to store information in a more computationally sophisticated and behaviorally relevant manner (Frémaux and Gerstner 2016).

In vitro studies investigating the role of NE in synaptic plasticity have primarily used stimulation protocols that induce standard Hebbian LTP, such as bouts of high‐frequency synaptic stimulation or repeated pairing of pre‐ and postsynaptic action potentials (O'Dell et al. 2015; Brzosko et al. 2019). Growing evidence indicates, however, that a non‐Hebbian form of LTP, known as behavioral timescale synaptic plasticity (BTSP), has a key role in hippocampus‐dependent spatial learning (Bittner et al. 2017; Priestley et al. 2022; Grienberger and Magee 2022; Zhao et al. 2022; Madar et al. 2025). In BTSP, dendritic plateau potentials serve as instructive signals that enable LTP induction at synapses activated seconds before or after plateau potentials (Bittner et al. 2017; Xiao et al. 2023). Dendritic plateau potentials also trigger somatic complex‐spike (CS) bursts in CA1 pyramidal cells (Takahashi and Magee 2009; Grienberger et al. 2014), and a CS burst‐dependent form of LTP in CA1 pyramidal cells has non‐Hebbian properties like those seen in BTSP (O'Dell 2022). Given the information encoded by LC neurons, NE likely has an important role in BTSP. Consistent with this notion, activation of LC axons in the hippocampus regulates the formation of place‐specific firing in CA1 pyramidal cells, presumably through modulation of BTSP (Kaufman et al. 2022). Moreover, β‐AR activation facilitates the induction of CS burst‐dependent LTP (Thomas et al. 1996; Winder et al. 1999). Beyond this, however, little is known about the effects of β‐AR activation on the properties of BTSP and CS burst‐dependent LTP.

Recent findings indicate that the induction of CS burst‐dependent LTP is regulated by multiple forms of heterosynaptic plasticity (O'Dell 2022). For instance, brief trains of theta‐frequency synaptic stimulation that induce modest homosynaptic potentiation also induce a heterosynaptic facilitation of EPSP‐evoked CS bursting at other synapses that enables asynchronously activated synapses to interact in a cooperative fashion and undergo LTP (O'Dell 2022). In contrast, EPSP‐evoked CS bursting elicited during longer trains of theta‐frequency stimulation induces robust homosynaptic potentiation but also triggers a form of synaptic competition that strongly inhibits LTP induction at other synapses (O'Dell 2022). Given the crucial role of cooperativity and competition in CS burst‐dependent LTP, the heterosynaptic forms of plasticity underlying these processes may provide key targets where NE can act to regulate CS burst‐dependent changes in synaptic strength. Thus, in this study I investigated how β‐AR activation affects synaptic interactions during the induction of CS burst‐dependent LTP.

## 2 Methods

### 2.1 Animals and Slice Preparation

Hippocampal slices from the dorsal hippocampus were obtained from 8 to 14 weeks old, male and female C57Bl/6 mice (#027 Charles River Laboratories). Mice were deeply anesthetized with isoflurane and, following cervical dislocation, the brain was removed and placed in cold (~4°C), oxygenated (95% O_2_/5% CO_2_) ACSF containing 124 mM NaCl, 4 mM KCl, 25 mM NaHCO_3_, 1 mM NaH_2_PO_4_, 2 mM CaCl_2_, 1.2 mM MgSO_4_, and 10 mM glucose. Hippocampi were then dissected from the brain and 400‐μm‐thick slices were prepared using a manual tissue slicer. The CA3 region was removed, and slices were then transferred into interface‐type chambers continuously perfused with ACSF (2–3 mL/min) and allowed to recover (at 30°C) for at least 2 h before recordings. All techniques were approved by the Institutional Animal Care and Use Committee at the University of California, Los Angeles, and were done in compliance with U.S. Public Health Service guidelines.

### 2.2 Electrophysiological Recordings

**FIGURE 1:**
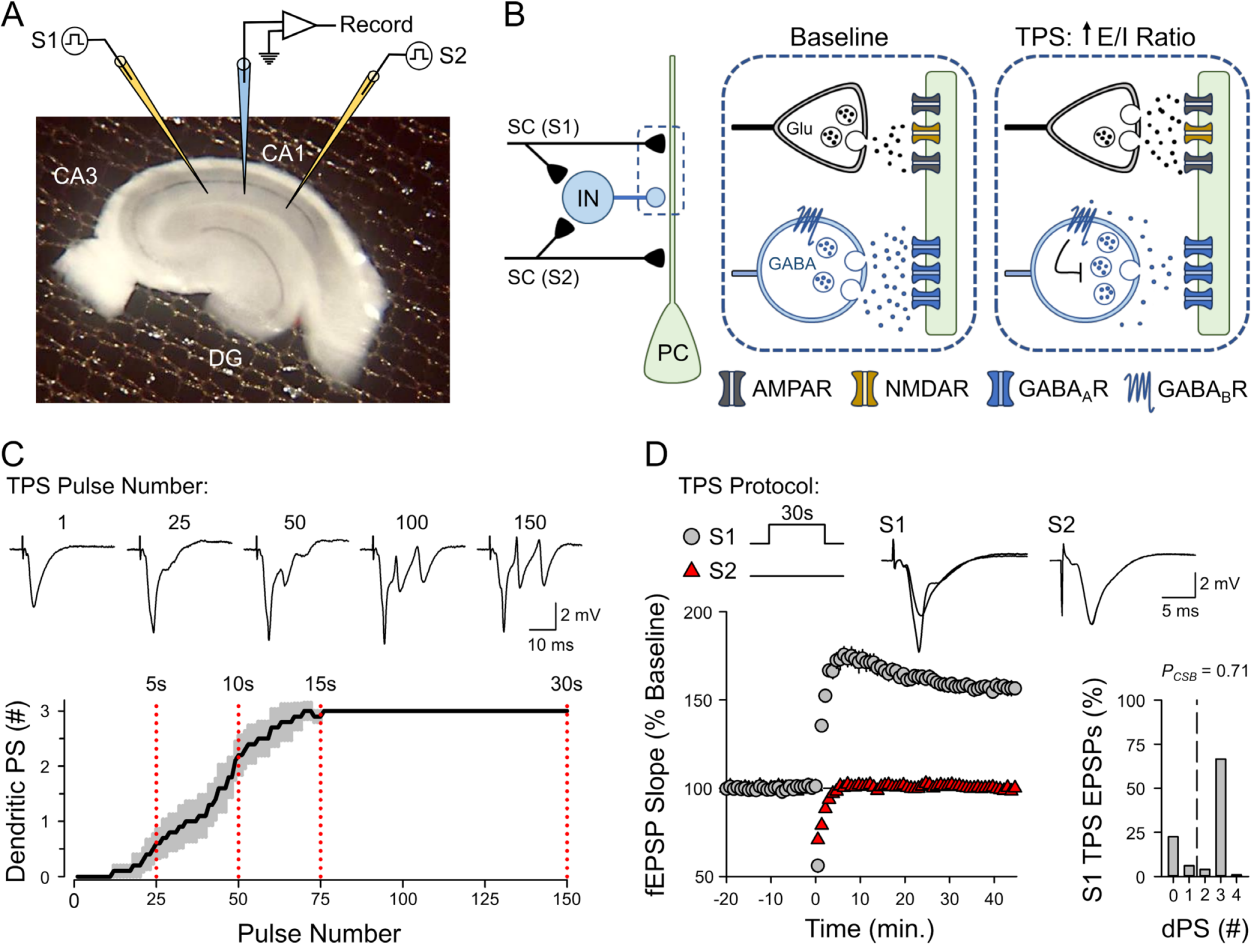
Theta‐pulse stimulation (TPS) triggers EPSP‐evoked CS bursting and induces LTP. (A) Picture shows hippocampal slice as well as placement of stimulating electrodes (S1 and S2) and recording electrode in stratum radiatum of the CA1 region. (B) Model depicting short‐term changes in excitatory and inhibitory transmission elicited by TPS. *Left*: Simplified circuit mediating feedforward inhibition in the CA1 region. Schaffer collateral (SC) fiber inputs onto CA1 pyramidal cells (PC) also activate GABAergic interneurons (IN) that inhibit CA1 pyramidal cells. Different groups of SC fibers (S1 and S2) activate a common pool of interneurons. *Middle*: Feedforward inhibition elicited by activation of S1 SC fibers during baseline synaptic stimulation opposes EPSP‐evoked spiking. *Right*: During S1 TPS, excitatory transmission at S1 synapses is facilitated (Babiec et al. 2017) while activation of presynaptic GABA_B_ receptors on inhibitory interneurons suppresses feedforward inhibition (Davis et al. 1991; Mott and Lewis 1991). The resulting increase in the ratio of excitatory to inhibitory synaptic strength (E/I ratio) enables EPSP‐evoked CS bursting. Note that because S1 and S2 inputs activate a common pool of inhibitory neurons, the suppression of feedforward inhibition induced by S1 TPS will also enhance postsynaptic responses elicited by S2 synapses. (C) Number of dendritic population spikes (dPS) evoked by EPSPs during a 30‐s‐long train of TPS. Shading represents ± SEM (*n* = 10). Traces show fEPSPs evoked by the indicated pulse numbers during TPS. (D) LTP induced by 30 s of TPS delivered to S1 synapses at time = 0. Results are from the same experiments shown in C. Traces show superimposed fEPSPs elicited by S1 and S2 stimulation during baseline and 45 min post TPS. Histogram shows the number of EPSPs evoking 0–4 dPSs during TPS from all experiments.

Extracellular recordings were done using slices maintained at 30°C in an interface‐type recording chamber perfused with ACSF (Figure 1A). Two bipolar, nickel‐chromium wire stimulating electrodes were placed in stratum radiatum to activate independent groups of Schaffer collateral/commissural fiber synapses onto CA1 pyramidal cells (hereafter referred to as S1 and S2 synapses). A glass microelectrode filled with ACSF (resistance = 10 MΩ) was placed in stratum radiatum to record field excitatory postsynaptic potentials (fEPSPs). Signals were acquired with a Multi‐Clamp 700B amplifier (Molecular Devices), low pass filtered at 2 kHz and digitized at 10 kHz. fEPSPs were evoked by capacity‐coupled voltage pulses (0.02 ms duration) delivered by a Grass S88 stimulator. After determining the maximal amplitude of fEPSPs evoked by each stimulating electrode, the stimulation intensity was adjusted to evoke fEPSPs with an amplitude of approximately 50% of the maximal amplitude. Standard techniques were used to ensure activation of independent groups of synapses (O'Dell 2022), and alternating pulses of S1 and S2 presynaptic fiber stimulation were then delivered at 0.04 Hz. All chemicals were obtained from Millipore‐Sigma except for tetrodotoxin (TTX) (Alomone Labs) and gabazine (Abcam). Stock solutions of the β‐AR agonist isoproterenol (ISO) were prepared fresh daily (1 mM in dH_2_O). Stock solutions of gabazine (1 mM), TTX citrate (1 mM), and (S)‐(‐)‐propranolol hydrochloride (5 mM) were prepared in dH_2_O and stored at −20°C. Stock solutions of 8‐cyclopentyl‐1,3‐dipropylxanthine (DPCPX, 2 mM) were prepared in DMSO and stored at −20°C.

In interface‐type recording chambers, hippocampal slices rest on a nylon mesh, with the bottom portion of the slice in contact with ACSF and the top exposed to a humidified atmosphere consisting of 95% O_2_ and 5% CO_2_ (Figure 1A). Compared to recording chambers where slices are fully immersed in ACSF, the enhanced oxygenation provided by interface chambers significantly improves the long‐term viability of healthy tissue (Gray and O'Dell 2013). However, a limitation of interface chambers is the slow diffusion of bath‐applied drugs into and out of the slice, as only part of the tissue is directly exposed to ACSF. To account for this, ISO was bath‐applied for 10 min, a duration sufficient for the facilitation of fEPSP slopes induced by β‐AR activation to reach a steady state.

### 2.3 CS Burst‐Dependent LTP Induction Protocol

CS burst‐dependent LTP was induced using theta‐pulse stimulation (TPS) protocols (single pulses of presynaptic fiber stimulation delivered at 5 Hz). Like more commonly used theta‐burst stimulation (TBS) protocols (Larson and Munkácsy 2015), TPS uses 5 Hz stimulation to mimic the ~5–10 Hz theta frequency modulation of CA1 pyramidal cell activity observed in vivo (Colgin 2016). However, unlike TBS, where high‐frequency bursts are delivered at 5 Hz, TPS uses single stimulation pulses. TPS induces a facilitation of excitatory synaptic transmission and a depression of feedforward, GABAergic inhibition (Davis et al. 1991; Mott and Lewis 1991; Babiec et al. 2017) (Figure 1B). Together, these short‐term forms of plasticity interact to produce a pronounced shift in the balance of excitatory and inhibitory transmission that allows EPSPs to escape the effects of feedforward inhibition and elicit postsynaptic CS bursts (McCarren and Alger 1985; Mott and Lewis 1991; Babiec et al. 2017). Notably, memory engram cells in the CA1 region exhibit repetitive, theta‐frequency bursting during memory encoding (Tanaka et al. 2018). Theta‐frequency CS bursting in CA1 pyramidal cells is also prominent during learning on odor discrimination and spatial learning tasks (Otto et al. 1991). Thus, TPS reproduces, in vitro, the theta‐frequency modulated CS bursting observed in CA1 pyramidal cells during memory encoding in vivo.

### 2.4 Statistical Analysis

Average slopes of fEPSPs (normalized to baseline) recorded 40–45 min post‐TPS were used for statistical comparisons. EPSP‐evoked CS bursting during TPS was quantified by visually inspecting fEPSPs evoked during TPS and counting the number of negative‐going population spikes (PSs) elicited by each EPSP during the train. CS bursts were defined as fEPSPs containing 2 or more PSs. The probability of EPSP‐evoked CS bursting (*P*_CSB_), determined from the number of EPSPs eliciting CS bursts relative to the total number of EPSPs evoked during TPS, was used for statistical comparisons. Heterosynaptic depression was measured using the slope of the first S2 fEPSP (normalized to baseline) elicited after a train of TPS delivered to S1 synapses. No obvious sex differences were found, and results from male and female mice were combined. Student's *t*‐tests or, where appropriate, Mann–Whitney Rank Sum tests were used to evaluate statistical significance between two groups. Correlations were determined using Pearson Product Moment Correlation tests. Multiple comparisons were done using one‐way or two‐way ANOVAs with Student–Newman–Keuls (SNK) *post hoc* comparisons or Kruskal‐Wallis one‐way ANOVA on Ranks followed by Dunn's tests for multiple, pairwise comparisons. Data were collected and analyzed using pClamp10 software (Molecular Devices). Statistical tests were performed using SigmaPlot 12.5 (Grafiti). Results are reported as mean ± SEM, and full results of statistical tests are provided in the figure legends.

## 3 Results

### 3.1 Distinct Patterns of TPS Reveal Cooperative and Competitive Synaptic Interactions Regulating CS Burst‐Dependent LTP Induction

**FIGURE 2:**
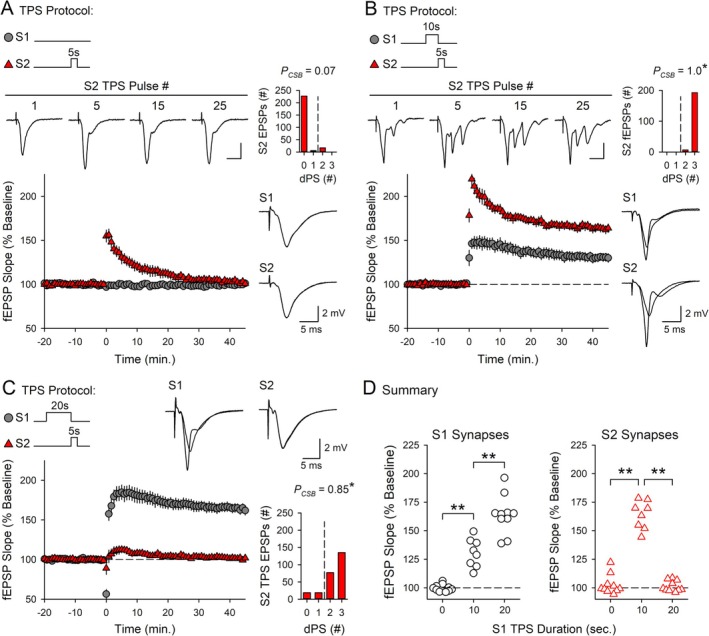
Cooperative and competitive synaptic interactions regulate CS burst‐dependent LTP induction. (A) 5 s of TPS delivered to S2 synapses alone (at time = 0) failed to elicit CS bursting and had no lasting effect on synaptic strength (*n* = 10). (B) 10 s of TPS delivered to S1 synapses before 5 s of S2 TPS facilitate LTP induction at S2 synapses (*n* = 8). Traces in A and B show fEPSPs evoked by the indicated pulses during S2 TPS (top, calibration bars are 2 mV and 10 ms) and superimposed fEPSPs elicited by S1 and S2 stimulation during baseline and 45 min post‐TPS (right). (C) 20 s of TPS delivered to S1 synapses before 5 s of S2 TPS inhibits LTP induction at S2 synapses (*n* = 10). Traces show superimposed fEPSPs evoked by S1 and S2 stimulation during baseline and 45 min post‐S2 TPS. Histograms in A–C show the number of EPSPs evoking 0–3 dendritic PSs during S2 TPS. *p < 0.05 compared to *P*_CSB_ during 5 s of TPS delivered to S2 synapses alone, one‐way ANOVA on Ranks with Dunn's *post hoc* tests, *H*_(2)_ = 21.363, *p* < 0.001. (D) S1 (left) and S2 (right) fEPSP slopes 45 min after S2 TPS from all experiments in A–C. Changes in synaptic strength at S1 and S2 synapses were analyzed separately using one‐way ANOVAs with SNK *post hoc* comparisons (**p < 0.001, S1 synapses: *F*_(2,25)_ = 68.107, *p* < 0.001; S2 synapses: *F*_(2,25)_ = 140.264, *p* < 0.001).

Because EPSP‐evoked CS bursting, synaptic cooperativity, and synaptic competition are recruited by very different patterns of TPS, the properties of CS burst‐dependent LTP are strongly dependent on the duration of TPS trains (O'Dell 2022). This can be seen in the results shown in Figures 1 and 2, which highlight four key properties of CS burst‐dependent LTP induced by TPS trains lasting 5 to 30 s. First, during TPS, CA1 pyramidal cells transition from single spike firing to burst firing after approximately 7–10 s of stimulation (Figure 1C). A previous study using somatic whole‐cell current‐clamp recordings demonstrated that these EPSP‐evoked bursts, which appear as multiple, negative‐going PSs in dendritic field potential recordings (Figure 1C, Video S1), exhibit many of the defining features of CS bursts recorded in vivo (O'Dell 2022). Second, because CS bursts provide the postsynaptic depolarization needed for NMDA receptor (NMDAR) activation during TPS (Thomas et al. 1998; O'Dell 2022), the induction of LTP by different durations of TPS reflects the activity dependence of EPSP‐evoked CS bursting. For example, 30 s of TPS delivered to S1 synapses elicited robust EPSP‐evoked CS bursting (*P*_CSB_ = 0.71, *n* = 10) and induced a synapse‐specific potentiation of S1 synapses (fEPSPs potentiated to 156% ± 4% of baseline) (Figure 1D). In contrast, a brief, 5‐s‐long train of TPS that ended before the onset of CS bursting (*P*_CSB_ = 0.07, *n* = 10) had no lasting effect on synaptic strength (45 min post‐TPS, fEPSPs were 102% ± 3% of baseline) (Figure 2A). Third, unlike conventional Hebbian induction protocols, where near synchronous coactivation of synaptic inputs is required for synapses to interact in a cooperative fashion and undergo LTP (Gustafsson and Wigström 1986; Lin et al. 2003), CS burst‐dependent LTP can be induced in a highly cooperative manner by sequential, asynchronous activation of independent groups of synapses (O'Dell 2022). An example of this is shown in Figure 2B, where 10 s of TPS was delivered to S1 synapses to bring CA1 pyramidal cells to threshold for EPSP‐evoked CS bursting before 5 s of TPS was delivered to S2 synapses. Although 5 s of TPS delivered to S2 synapses by itself elicits few CS bursts (Figure 2A), EPSP‐evoked CS bursting at S2 synapses was strongly enhanced when S2 TPS was delivered after 10 s of TPS delivered to S1 synapses (*P*_CSB_ during S2 TPS = 1.0, *n* = 8), and S2 fEPSPs potentiated to 164% ± 4% of baseline (Figure 2B). Due to the activity dependence of CS bursting during TPS (Figure 1C), relatively few of the EPSPs evoked during S1 TPS elicited CS bursts (*P*_CSB_ = 0.3), and S1 synapses potentiated to just 131% ± 4% of baseline (Figure 2B). Thus, although 10 s of TPS induces relatively modest homosynaptic LTP, it produces a powerful heterosynaptic facilitation of EPSP‐evoked bursting and LTP induction at S2 synapses. Indeed, the potentiation of S2 synapses induced by the S1 → S2 TPS protocol used in these experiments was 2‐fold larger than that seen at S1 synapses (*t*_(14)_ = 5.353, *p* = 1.02 × 10^−4^), even though the S2 TPS train was only half as long as the TPS train delivered to S1 synapses. Finally, TPS trains lasting 20 s or more trigger competitive synaptic interactions that suppress LTP at other inputs (O'Dell 2022). For example, increasing the duration of S1 TPS to 20 s significantly enhanced homosynaptic LTP at S1 synapses (S1 fEPSPs potentiated to 164% ± 5% of baseline, *n* = 10), but abolished the heterosynaptic facilitation of TPS‐induced LTP at S2 synapses (45 min post‐TPS, S2 fEPSPs were 101% ± 2% of baseline), even though EPSPs elicited CS bursts during S2 TPS (*P*_CSB_ = 0.85) (Figure 2C). Thus, longer trains of TPS that induce robust homosynaptic LTP also generate a winner‐take‐all form of synaptic competition that blocks LTP induction at other synapses (Figure 2D).

### 3.2 β‐AR Activation Enhances Competitive Synaptic Interactions During the Induction of CS Burst‐Dependent LTP

**FIGURE 3:**
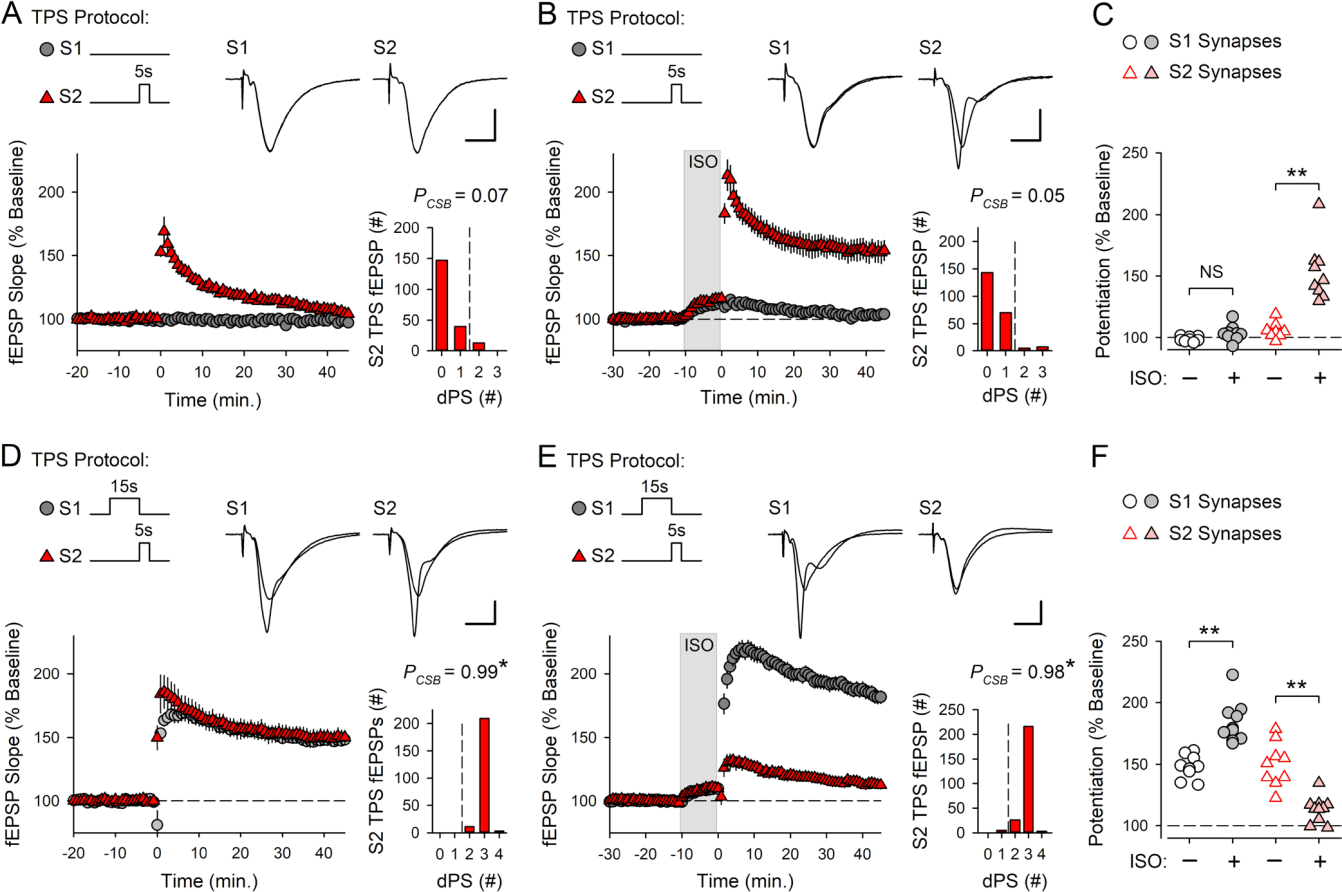
β‐AR activation enhances LTP and competitive synaptic interactions during the induction of CS burst‐dependent LTP. (A) Control experiments where 5 s of TPS was delivered to S2 synapses (at time = 0) (*n* = 8). (B) S2 TPS was delivered at the end of a 10‐min bath application of ISO (1.0 μM, indicated by the shaded region) (*n* = 9). (C) S1 and S2 fEPSP slopes 45 min after S2 TPS from all experiments in A and B. A two‐way ANOVA revealed a significant difference between S1 and S2 synapses (*F*_(1,30)_ = 38.962, *p* < 0.001), a significant effect of ISO (*F*_(1,30)_ = 33.212, *p* < 0.001), and a significant synapse × ISO interaction (*F*_(1,30)_ = 22.281, *p* < 0.001). ISO significantly enhanced LTP induction at S2 synapses (**p < 0.001) but had no lasting effect on S1 synapses (NS, not significant, *p* = 0.467, SNK *post hoc* tests). (D) Control experiments where 15 s of TPS was delivered to S1 synapses prior to S2 TPS (*n* = 9). (E) 15 s of S1 TPS before S2 TPS was delivered at the end of a 10‐min bath application of ISO (*n* = 10). (F) S1 and S2 fEPSP slopes 45 min post‐S2 TPS from all experiments in D and E. A two‐way ANOVA with SNK *post hoc* tests revealed a significant difference between S1 and S2 synapses (*F*_(1,34)_ = 55.723, *p* < 0.001) and a significant synapse × ISO interaction (*F*_(1,34)_ = 62.341, *p* < 0.001). ISO significantly enhanced LTP induction at S1 synapses and inhibited LTP induction at S2 synapses (**p < 0.001). Histograms show the number of EPSPs evoking 0–4 dendritic PSs during S2 TPS. EPSP‐evoked CS bursting during S2 TPS was significantly enhanced by prior S1 TPS (*p < 0.05, one‐way ANOVA on Ranks with Dunn's *post hoc* comparisons to control experiments shown in panel A, *H*_(3)_ = 30.874, *p* < 0.001). Traces show superimposed fEPSPs evoked by S1 and S2 stimulation during baseline and 45 min post‐S2 TPS (calibration bars are 2 mV and 5 ms).

The β‐AR antagonist propranolol had no effect on TPS‐induced LTP, indicating that β‐AR activation is not required for the induction of CS burst‐dependent LTP (Figure S1). Numerous studies have found, however, that β‐AR activation enhances TPS‐induced LTP (Thomas et al. 1996; Winder et al. 1999; Gelinas et al. 2008; Qian et al. 2012; Jami et al. 2023). Consistent with these findings, although 5 s of TPS delivered to S2 synapses had no lasting effect on synaptic transmission in control experiments (Figure 3A), S2 fEPSPs potentiated to 153% ± 8% of baseline (*n* = 9) when TPS was delivered at the end of a 10‐min bath application of 1.0 μM ISO (Figure 3B,C). To explore the effects of β‐AR activation on synaptic interactions during the induction of CS burst‐dependent LTP, I next examined the effects of ISO on the heterosynaptic facilitation of LTP induction at S2 synapses produced by a 15‐s‐long train of TPS delivered to S1 synapses. In control experiments, 15 s of TPS delivered to S1 synapses before a 5‐s‐long train of S2 TPS facilitated EPSP‐evoked CS bursting during S2 TPS (*P*_CSB_ = 0.99, *n* = 9) and enabled LTP induction at S2 synapses (S2 fEPSPs potentiated to 149% ± 6% of baseline) (Figure 3D). Surprisingly, although LTP induction at S1 synapses was enhanced when this pattern of S1 → S2 TPS was delivered in the presence of ISO, LTP induction at S2 synapses was strongly suppressed (45 min post‐TPS in ISO S2 fEPSPs were 113% ± 3% of baseline, *n* = 10) (Figure 3E,F). This paradoxical ability of ISO to simultaneously enhance LTP induction at S1 synapses and inhibit the induction of LTP at S2 synapses indicates that β‐AR activation modulates the heterosynaptic forms of plasticity underlying synaptic interactions during CS burst‐dependent LTP induction.

**FIGURE 4:**
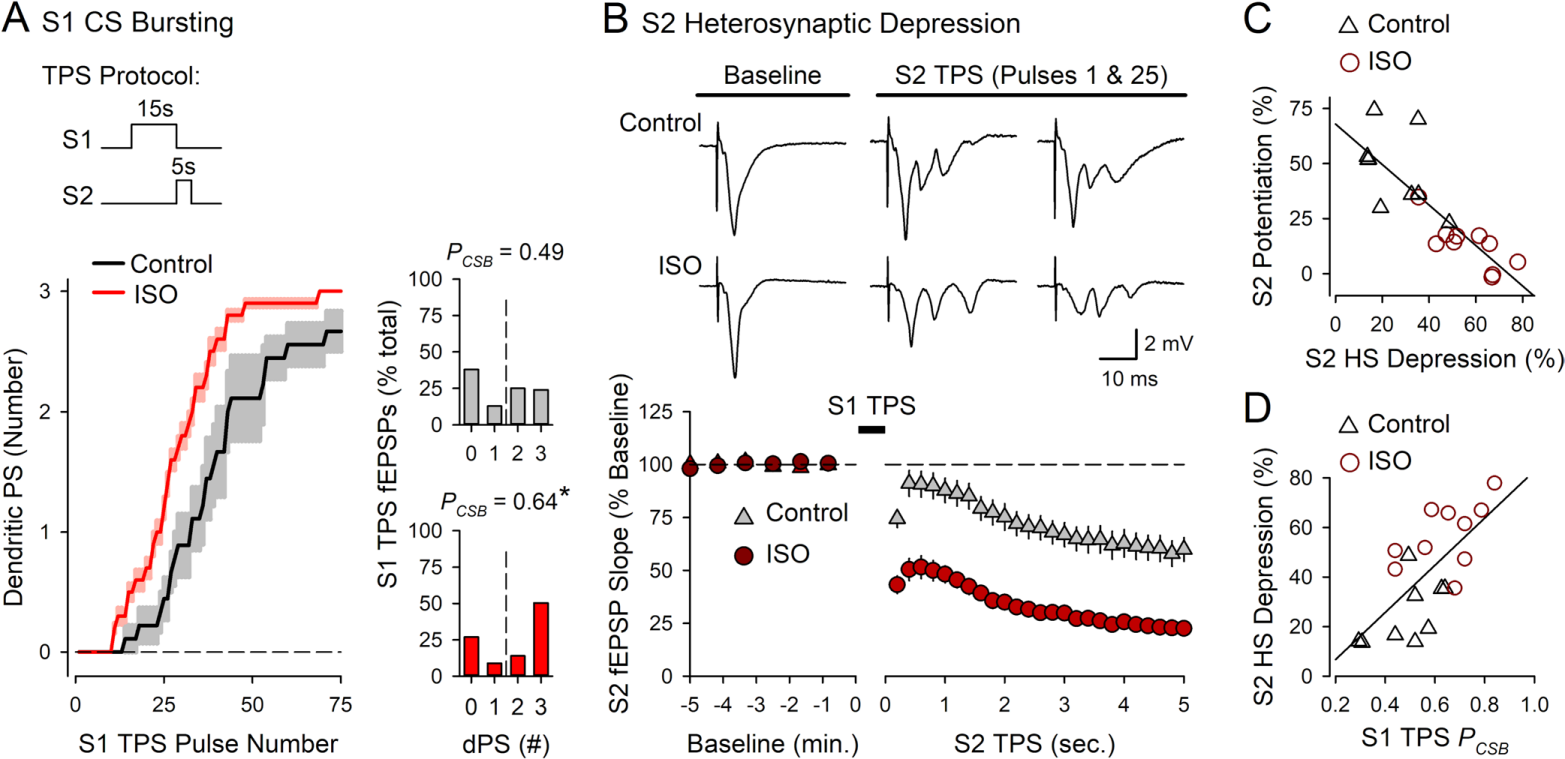
β‐AR activation enhances EPSP‐evoked CS bursting and TPS‐induced heterosynaptic depression. Results are from the experiments shown in Figure 3D,E. (A) Number of dendritic PSs evoked by EPSPs during 15 s of TPS delivered to S1 synapses in the absence (control) and presence of 1.0 μM ISO. Shading represents ± SEM. Histograms show the number of EPSPs eliciting 0–3 dendritic PSs during S1 TPS in control experiments (*P*_CSB_ = 0.490) and S1 TPS in the presence of ISO (*P*_CSB_ = 0.643, *t*_(17)_ = 2.538, *p = 0.0212). (B) fEPSP slopes during S2 TPS delivered after 15 s of S1 TPS in the absence and presence of ISO. Traces show S2 fEPSPs evoked before S1 TPS (baseline) and the first and last fEPSPs evoked during S2 TPS. (C, D) Scatter plots show correlations between S1 TPS‐induced heterosynaptic (HS) depression and LTP induction at S2 synapses (C) (*r* = −0.837, *p* = 7.75 × 10^−6^) and between the probability of EPSP‐evoked CS bursting during S1 TPS and the magnitude of heterosynaptic depression at S2 synapses (D) (*r* = 0.696, *p* = 9.4 × 10^−4^).

β‐AR activation had two prominent effects on postsynaptic responses evoked during S1 and S2 TPS that likely contribute to the bidirectional effects of ISO on LTP induction shown in Figure 3E,F. First, β‐AR activation increased EPSP‐evoked CS bursting during the 15 s of TPS delivered to S1 synapses (Figure 4A). Given the crucial role of EPSP‐evoked CS bursting in TPS‐induced LTP (Thomas et al. 1998; O'Dell 2022), the increase in CS bursting induced by β‐AR activation likely contributes to the ISO‐induced facilitation of LTP induction at S1 synapses, as suggested by others (Winder et al. 1999; Gelinas et al. 2008). Second, 15 s of S1 TPS in the presence of ISO induced a strong, heterosynaptic depression of transmission at S2 synapses that was maintained throughout the 5‐s‐long train of TPS delivered to S2 synapses (Figure 4B). Interestingly, there was a significant, negative correlation between the potentiation of S2 synapses and the amount of heterosynaptic depression induced by S1 TPS (Figure 4C). Moreover, the amount of heterosynaptic depression at S2 synapses was highly correlated with the probability of EPSP‐evoked CS bursting during S1 TPS (Figure 4D). Together, these correlations suggest that β‐AR activation facilitates synaptic competition and suppresses LTP induction at S2 synapses by enhancing a CS burst‐dependent form of heterosynaptic depression.

### 3.3 β‐AR Activation Enhances a CS Burst‐Dependent Form of Heterosynaptic Depression

**FIGURE 5:**
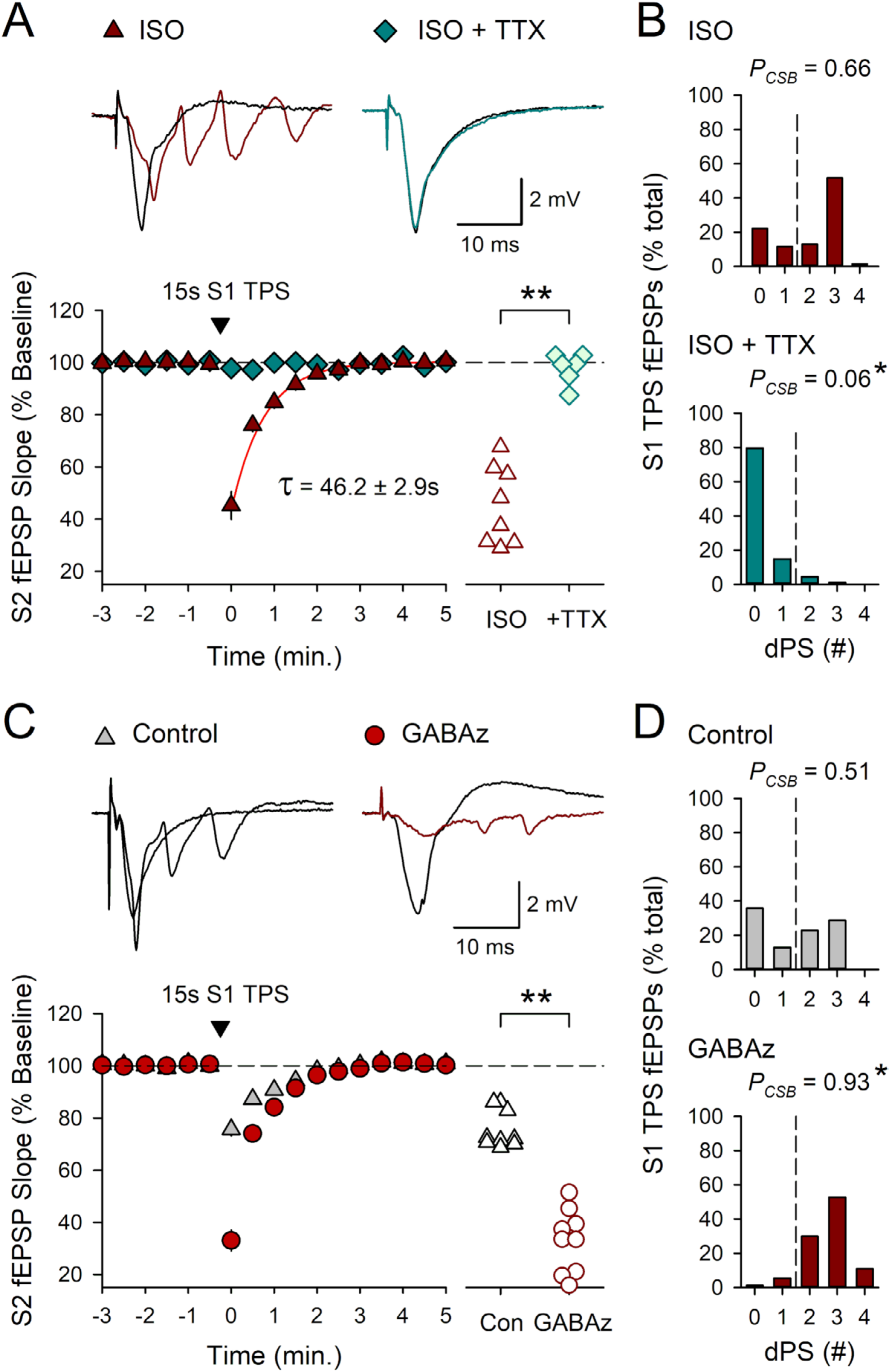
β‐AR activation enhances a CS burst‐dependent form of heterosynaptic depression. S2 synapses were activated at 0.033 Hz before and after a 15‐s‐long train of S1 TPS. (A) S1 TPS delivered at the end of a 10‐min bath application of 1.0 μM ISO (*n* = 8) or ISO plus 0.2 μM TTX (*n* = 6). Red line shows a single exponential fit to the recovery of S2 responses following S1 TPS‐induced heterosynaptic depression. Scatter plot shows slopes of first S2 fEPSPs evoked post‐S1 TPS from all experiments (*t*_(12)_ = 8.051, **p = 3.52 × 10^−6^). (B) Number of EPSPs eliciting 0–4 dendritic PSs during S1 TPS in the presence of ISO (*P*_CSB_ = 0.662) and ISO + TTX (*P*_CSB_ = 0.056, Mann–Whitney *U* = 0.0, *p < 0.001). (C) S1 TPS was delivered either alone (control, *n* = 9) or at the end of a 10‐min bath application of 0.5 μM gabazine (GABAz) (*n* = 9). Scatter plot shows slopes of first S2 fEPSPs evoked post‐S1 TPS from all experiments (*t*_(16)_ = 9.098, **p = 1.01 × 10^−7^). (D) Number of EPSPS eliciting 0–4 dendritic PSs during S1 TPS in control experiments (*P*_CSB_ = 0.514) and in the presence of gabazine (*P*_CSB_ = 0.934, Mann Whitney *U* = 2.5, *p < 0.001). Traces in A and C show superimposed S2 fEPSPs recorded before and after S1 TPS.

To directly test the notion that heterosynaptic depression is induced by postsynaptic CS bursting, I examined whether suppressing EPSP‐evoked CS bursting with a low concentration of the Na^+^ channel blocker TTX (Thomas et al. 1998; O'Dell 2022) inhibits TPS‐induced heterosynaptic depression. In these experiments, S2 synapses were activated at 0.033 Hz before and after a 15‐s‐long TPS train delivered to S1 synapses. In control experiments, 15 s of S1 TPS delivered at the end of a 10‐min bath application of 1.0 μM ISO induced strong EPSP‐evoked bursting (*P*_CSB_ = 0.66, *n* = 9) and a large, transient depression at S2 synapses (S2 fEPSPs were initially depressed to 45% ± 5% of baseline and recovered with a time constant of approximately 46 s) (Figure 5A,B). Consistent with the notion that β‐AR activation enhances a CS burst‐dependent form of heterosynaptic depression, CS bursting during S1 TPS was blocked (*P*_CSB_ during S1 TPS = 0.07, *n* = 6) and the heterosynaptic depression of S2 synapses was abolished when ISO was co‐applied with 200 nM TTX (the first S2 fEPSPs evoked after S1 TPS were 98% ± 2% of baseline) (Figure 5A,B). Notably, suppressing feedforward, GABAergic inhibition enhances EPSP‐evoked CS bursting in CA1 pyramidal cells (Lovett‐Barron et al. 2012; Babiec et al. 2017). Thus, as an additional test of the role of postsynaptic CS bursting in heterosynaptic depression, I examined whether heterosynaptic depression is enhanced when S1 TPS is delivered in the presence of the GABA_A_ receptor antagonist gabazine. In control experiments, 15 s of S1 TPS induced a relatively modest amount of EPSP‐evoked CS bursting (*P*_CSB_ = 0.51, *n* = 9) and a small heterosynaptic depression of transmission at S2 synapses (S2 fEPSPs were initially depressed to 76% ± 2% of baseline) (Figure 5C,D). In contrast, EPSP‐evoked CS bursting was significantly enhanced when S1 TPS was delivered at the end of a 10‐min bath application of 0.5 μM gabazine (*P*_CSB_ = 0.93) and, consistent with the notion that TPS induces a CS burst‐dependent form of heterosynaptic depression, the first S2 fEPSPs evoked post‐S1 TPS were reduced to just 33% ± 4% of baseline (*n* = 9) (Figure 5C,D).

**FIGURE 6:**
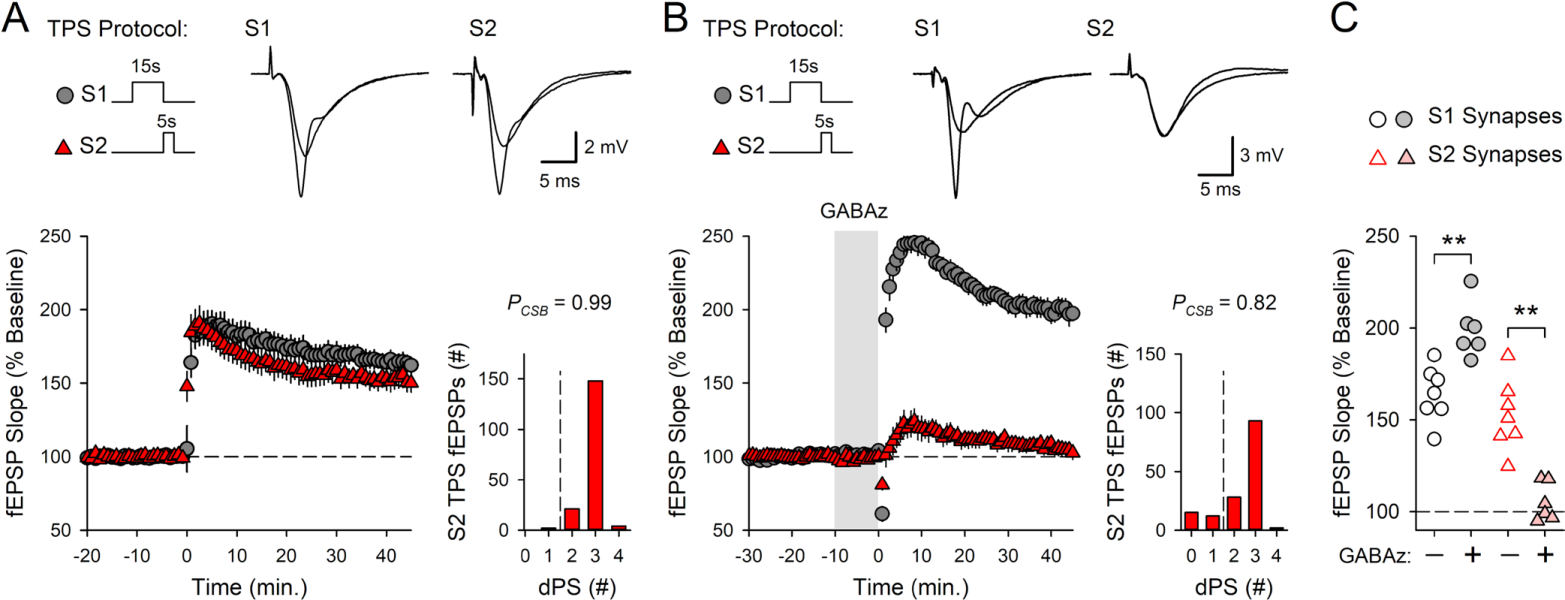
Suppressing GABAergic transmission enhances both LTP induction and synaptic competition. (A) Control experiments (*n* = 7) where a 15‐s‐long train of TPS was delivered to S1 synapses prior to 5 s of S2 TPS (at time = 0). (B) 15 s of S1 TPS before 5 s of S2 TPS was delivered at the end of a 10‐min bath application of 0.5 μM gabazine (GABAz) (*n* = 6). Histograms in A and B show the number of EPSPs evoking 0–4 dendritic PSs during S2 TPS, and traces show superimposed fEPSPs elicited by S1 and S2 stimulation during baseline and 45 min post‐S2 TPS. (C) S1 and S2 fEPSPs 45 min after S2 TPS from all experiments. A two‐way ANOVA with SNK *post hoc* tests revealed a significant difference between S1 and S2 synapses (*F*_(1,22)_ = 74.892, *p* < 0.001) and a significant synapse × gabazine interaction (*F*_(1,22)_ = 45.834, *p* < 0.001). Gabazine significantly enhanced LTP at S1 synapses and suppressed LTP induction at S2 synapses (**p < 0.001).

GABAergic inhibitory synaptic transmission opposes the induction of Hebbian LTP (Wigström and Gustafsson 1984), and GABA_A_ receptor antagonists are thus often used to facilitate LTP induction in in vitro studies of synaptic plasticity, including BTSP (Bittner et al. 2017; Xiao et al. 2023). The hypothesis that heterosynaptic depression underlies synaptic competition predicts, however, that the robust increase in TPS‐induced heterosynaptic depression induced by gabazine should have the opposite effect and inhibit, rather than enhance, the induction of LTP by cooperative synaptic interactions during TPS. To test this prediction, I examined the effects of gabazine on the heterosynaptic facilitation of LTP induction at S2 synapses produced by prior S1 TPS. As expected, in control experiments, 15 s of S1 TPS delivered prior to 5 s of S2 TPS induced LTP at S1 synapses (S1 fEPSPs potentiated to 164% ± 6% of baseline, *n* = 7) and enabled LTP induction at S2 synapses (S2 fEPSPs potentiated to 152% ± 7% of baseline) (Figure 6A). However, when this pattern of S1 → S2 TPS was delivered at the end of a 10‐min bath application of gabazine, the induction of LTP at S1 synapses was enhanced (S1 fEPSPs potentiated to 199% ± 6% of baseline, *n* = 6), and LTP induction at S2 synapses was abolished (45 min post‐TPS, S2 fEPSPs were 105% ± 4% of baseline) (Figure 6B,C). Thus, consistent with the notion that synaptic competition is mediated by a CS burst‐dependent form of heterosynaptic depression, the increase in S1 TPS‐induced CS bursting and heterosynaptic depression induced by gabazine is associated with a strong increase in synaptic competition that inhibits LTP induction at S2 synapses.

### 3.4 Synaptic Competition Is Mediated by a β‐AR‐Modulated and Adenosine Receptor‐Dependent Form of Heterosynaptic Depression

**FIGURE 7:**
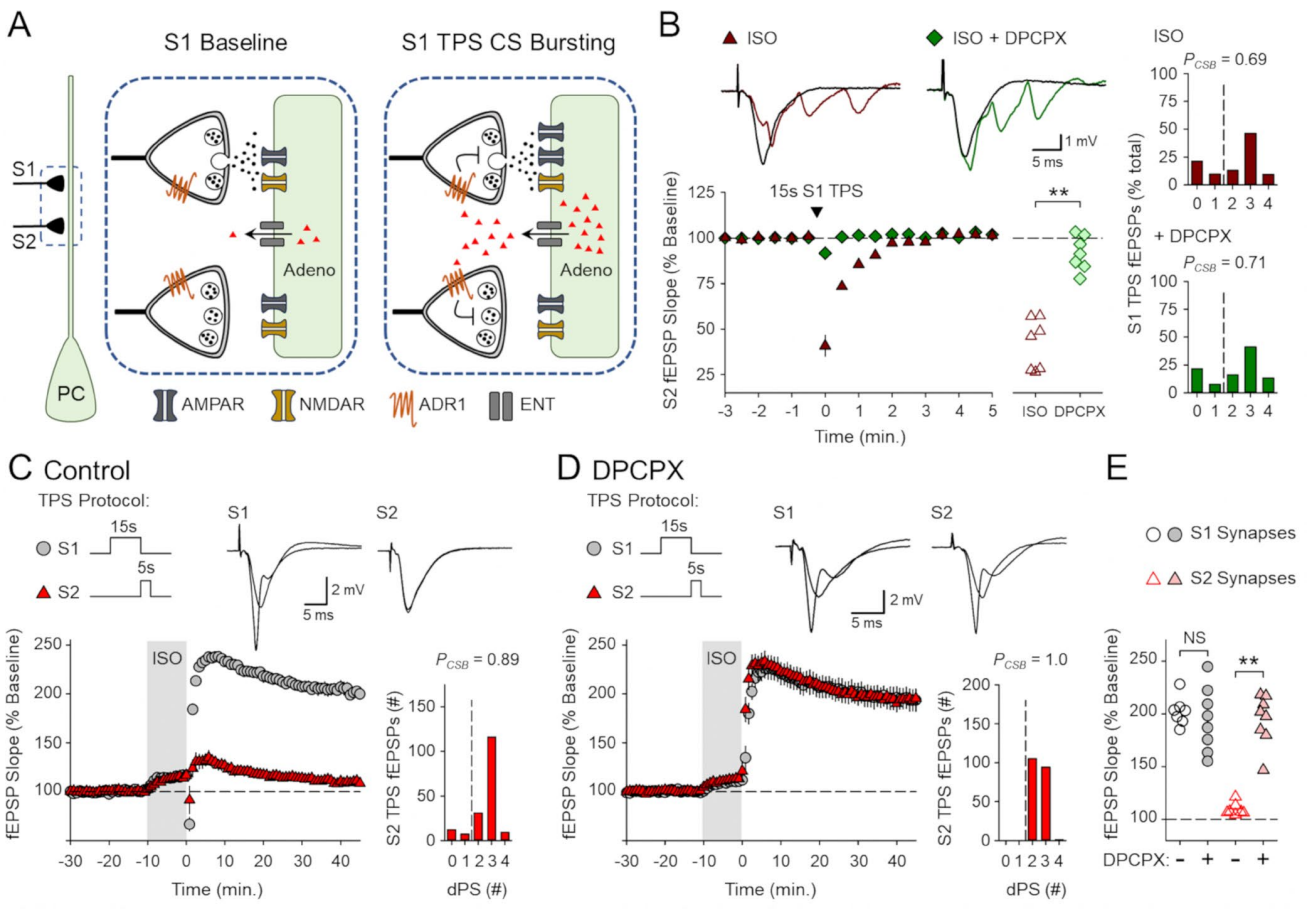
Synaptic competition is mediated by an A1 adenosine receptor‐dependent form of heterosynaptic depression. (A) Potential mechanism underlying TPS‐induced heterosynaptic depression. *Left*: S1 and S2 synaptic inputs onto postsynaptic CA1 pyramidal cell (PC). The modest release of adenosine (Adeno) during baseline stimulation of S1 synapses (middle) is dramatically enhanced by CS bursting during S1 TPS (right). Although EPSP‐evoked CS bursting induces LTP at S1 synapses (indicated by increased AMPAR content), it also triggers the release of adenosine via equilibrative nucleoside transporters (ENT) (Wu et al. 2023). Activation of presynaptic A1 adenosine receptors (ADR1) transiently inhibits transmission at S2 (and S1 synapses, see Figure 1D). (B) S2 synapses were activated at 0.033 Hz before and after a 15‐s‐long train of S1 TPS (delivered at time = 0 after a 10‐min bath application of 1.0 μM ISO). The heterosynaptic depression induced by S1 TPS in control experiments (ISO alone, *n* = 7) was inhibited when S1 TPS was delivered in the presence of ISO plus 400 nM DPCPX (*n* = 7). Scatter plot shows slopes of first S2 fEPSPs evoked post‐S1 TPS from all experiments (*t*_(12)_ = 7.875, **p = 4.42 × 10^−6^). Histograms show the number of EPSPs eliciting 0–4 dendritic PSs during S1 TPS in the presence of ISO (*P*_CSB_ = 0.69) and in the presence of ISO plus DPCPX (*P*_CSB_ = 0.71, *t*_(12)_ = 0.287, *p* = 0.779). Traces show superimposed S2 fEPSPs recorded during baseline and after S1 TPS. (C) Control experiments where 15 s of TPS delivered to S1 synapses before 5 s of S2 TPS was delivered at the end of a 10‐min bath application of 1.0 μM ISO (*n* = 7). (D) 15 s of S1 TPS before 5 s of S2 was delivered at the end of a 10‐min bath application of 1.0 μM ISO in slices continuously bathed in ACSF containing 400 nM DPCPX (*n* = 8). Traces in C and D show superimposed fEPSPs elicited by S1 and S2 stimulation during baseline and 45 min post‐S2 TPS. Histograms in C and D show the number of EPSPs evoking 0–4 dendritic PSs during S2 TPS. (E) Plot shows fEPSP slopes recorded 45 min post‐S2 TPS from all experiments in C and D. A two‐way ANOVA with SNK *post hoc* tests revealed a significant difference between S1 and S2 synapses (*F*_(1,26)_ = 35.706, *p* < 0.001), a significant effect of DPCPX (*F*_(1,26)_ = 24.628, *p* < 0.001), and a significant synapse × DPCPX interaction (*F*_(1,26)_ = 35.864, *p* < 0.001). DPCPX had no effect on LTP induction at S1 synapses (NS, *p* = 0.475) but significantly enhanced LTP induction at S2 synapses (**p < 0.001).

How might a CS burst‐dependent form of heterosynaptic depression give rise to competitive synaptic interactions during the induction of CS burst‐dependent LTP? One prominent form of heterosynaptic depression at excitatory synapses onto CA1 pyramidal cells is due to an inhibition of glutamate release caused by activation of presynaptic, A1‐type adenosine receptors (Grover and Teyler 1993; Mitchell et al. 1993; Wu and Saggau 1994). Moreover, bursts of postsynaptic action potentials trigger adenosine release from CA1 pyramidal cells' dendrites (Lovatt et al. 2012; Wu et al. 2023). Thus, synaptic competition induced by S1 TPS in the presence of ISO may be mediated by a presynaptic, adenosine receptor‐dependent form of heterosynaptic depression that, by inhibiting glutamate release, prevents the strong activation of postsynaptic NMDARs needed for LTP induction at S2 synapses (Figure 7A). To test this hypothesis, I first examined the effects of the A1 adenosine receptor antagonist DPCPX on the heterosynaptic depression induced by TPS. Consistent with the notion that TPS‐induced heterosynaptic depression is due to activation of A1 receptors, the heterosynaptic depression induced by 15 s of S1 TPS in the presence of ISO (S2 fEPSPs were initially reduced to 42% ± 5% of baseline, *n* = 7) was significantly inhibited when S1 TPS was delivered in the presence of ISO plus 400 nM DPCPX (the first S2 fEPSPs elicited post‐S1 TPS were 92% ± 4% of baseline, *n* = 7) (Figure 7B). To explore the role of heterosynaptic depression in synaptic competition, I next examined the effects of DPCPX on LTP induction when 15 s of S1 TPS was delivered before 5 s of S2 TPS in the presence of ISO. In control experiments, 15 s of S1 TPS induced LTP at S1 synapses (fEPSPs potentiated to 202% ± 5% of baseline, *n* = 7) and suppressed LTP induction at S2 synapses (45 min post‐TPS, S2 fEPSPs were just 110% ± 2% of baseline) (Figure 7C). In contrast, when this same pattern of S1 → S2 TPS was delivered in the presence of ISO plus DPCPX, S1 synapses potentiated to 194% ± 11% of baseline and S2 synapses potentiated to 194% ± 8% of baseline (*n* = 8) (Figure 7D). Thus, although DPCPX had no effect on LTP induction at S1 synapses, it blocked the enhancement of synaptic competition induced by β‐AR activation, thereby enabling LTP induction at S2 synapses (Figure 7E). DPCPX did not, however, enable the induction of LTP when 5 s of TPS was delivered to S2 synapses without prior S1 TPS (45 min post S2 TPS fEPSPs were 104% ± 2% of baseline, *n* = 8, data not shown). This indicates that blocking A1 receptors does not simply enhance LTP induction but instead selectively disrupts the increase in synaptic competition induced by β‐AR activation.

### 3.5 β‐AR Activation Enhances Cooperative Synaptic Interactions During the Induction of CS Burst‐Dependent LTP

**FIGURE 8:**
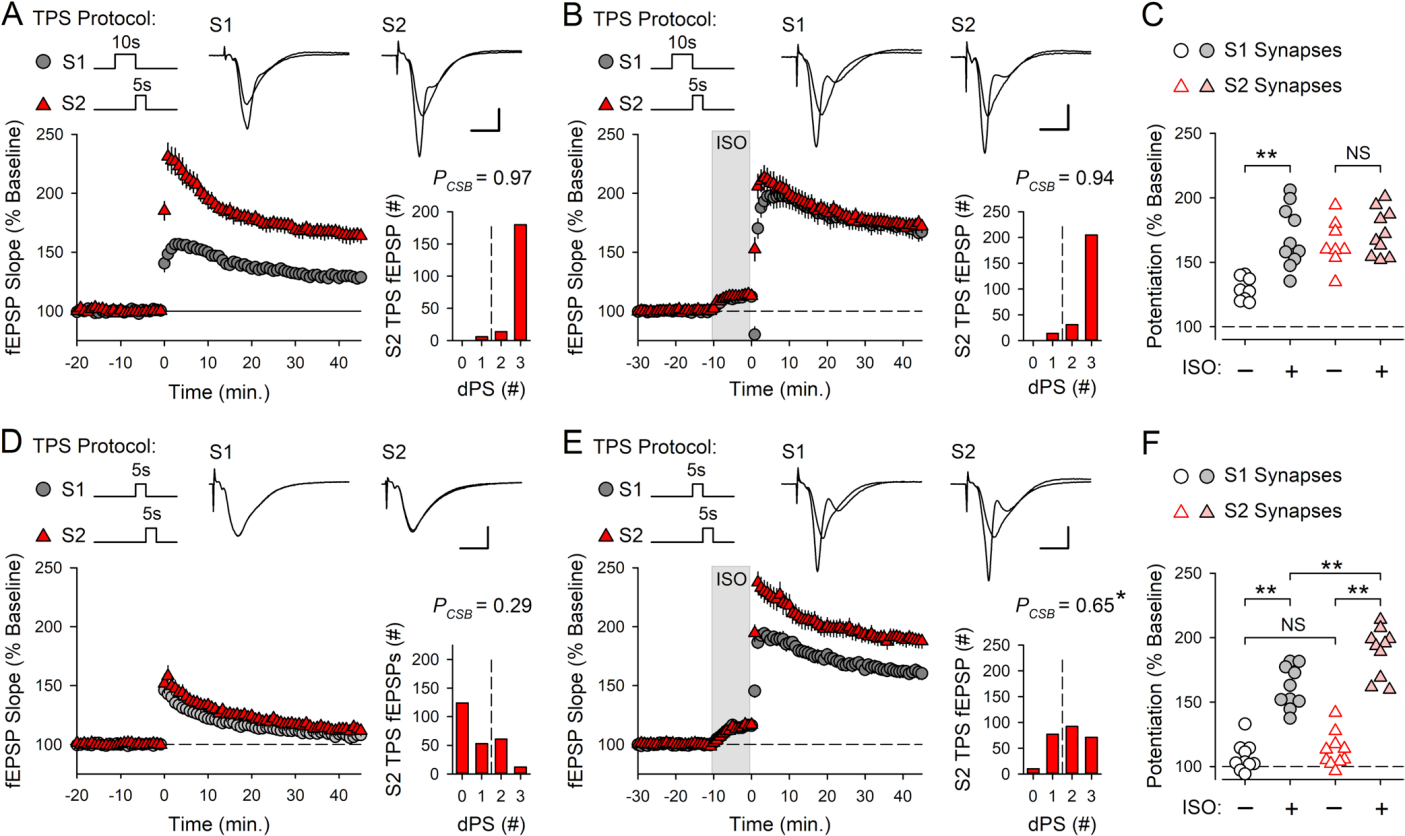
β‐AR activation induces an activity‐dependent facilitation of cooperative synaptic interactions during CS burst‐dependent LTP induction. (A) Control experiments (*n* = 8) where 10 s of TPS was delivered to S1 synapses before 5 s of S2 TPS (at time = 0). (B) 10 s of S1 TPS before S2 TPS was delivered at the end of a 10‐min bath application of ISO (*n* = 10). (C) S1 and S2 fEPSP slopes 45 min after S2 TPS from all experiments in A and B. Results were analyzed using two‐way ANOVA with SNK *post hoc* comparisons (**p < 0.001, NS, not significant, *p* = 0.679). There was a significant difference between S1 and S2 synapses (*F*_(1,32)_ = 9.906, *p* = 0.004), a significant effect of ISO (*F*_(1,32)_ = 15.516, *p* < 0.001), and a significant synapse × ISO interaction (*F*_(1,32)_ = 6.712, *p* = 0.014). (D) Control experiments where 5 s of S1 TPS was delivered before 5 s of S2 TPS (*n* = 10). (E) 5 s of S1 TPS before S2 TPS was delivered in the presence of ISO (*n* = 10). (F) S1 and S2 fEPSP slopes 45 min after S2 TPS from all experiments in D and E. A two‐way ANOVA with SNK post hoc comparisons (**p < 0.001, NS, *p* = 0.439) revealed a significant difference between S1 and S2 synapses (*F*_(1,36)_ = 11.626, *p* = 0.002), a significant effect of ISO (*F*_(1,36)_ = 181.462, *p* < 0.001), and a significant synapse × ISO interaction (*F*_(1,36)_ = 5.307, *p* = 0.027). Histograms show number of EPSPs evoking 0–3 CS bursts during S2 TPS in all experiments. CS bursting during S2 TPS was significantly enhanced by ISO (Mann Whitney *U* = 24.0, *p = 0.049). Traces show superimposed fEPSPs elicited by S1 and S2 stimulation during baseline and 45 min after S2 TPS.

Although delivering 15 s of TPS to S1 synapses before 5 s of S2 TPS induces similar amounts of LTP at both synapses in control experiments (Figures 3D and 6A), the enhancement of synaptic competition induced by β‐AR activation results in a selective potentiation of the more strongly activated S1 synapses (Figures 3E and 7C). This differential potentiation of more strongly activated synapses when TPS is delivered in the presence of ISO raises an intriguing question—how does β‐AR activation regulate synaptic interactions and LTP induction when S1 and S2 synapses are activated by more similar or even identical trains of TPS? To address this question, I examined how β‐AR activation influences LTP induction at S1 and S2 synapses when the duration of TPS delivered to S1 synapses (before 5 s of S2 TPS) was reduced to 10 s. Consistent with the results shown in Figure 2B, in control experiments, 10 s of S1 TPS induced a modest potentiation at S1 synapses (S1 fEPSPs potentiated to 129% ± 3% of baseline, *n* = 8) and enabled LTP induction at S2 synapses (S2 fEPSPs potentiated to 164% ± 6% of baseline) (Figure 8A). However, when this pattern of S1 → S2 TPS was delivered in the presence of ISO, β‐AR activation significantly enhanced LTP induction at S1 synapses (S1 fEPSPs potentiated to 169% ± 7% of baseline, *n* = 10) but had no effect on LTP induction at S2 synapses (S2 fEPSPs potentiated to 172% ± 6% of baseline) (Figure 8B,C). The enhancement of synaptic competition induced by β‐AR activation thus appears to be highly activity‐dependent. Indeed, β‐AR activation significantly enhanced LTP induction at both S1 and S2 synapses when the duration of S1 TPS was further reduced to 5 s (Figure 8D–F). In control experiments, 5 s of S1 TPS delivered before 5 s of S2 TPS had no lasting effect on synaptic transmission at S1 synapses and did not enable LTP induction at S2 synapses (45 min post‐TPS S1 and S2 synapses were 107% ± 4% and 113% ± 4% of baseline, respectively, *n* = 10) (Figure 8D). In the presence of ISO, this pattern of S1 → S2 TPS induced LTP at both S1 and S2 synapses and, surprisingly, the potentiation at S2 synapses was significantly larger than that seen at S1 synapses (S1 and S2 synapses potentiated to 161% ± 5% and 189% ± 6% of baseline, respectively, *n* = 10) (Figure 8E,F). Moreover, although 5 s of S1 TPS induced a modest heterosynaptic facilitation of EPSP‐evoked CS bursting at S2 synapses in control experiments (*P*_CSB_ = 0.29) (Figure 8D), EPSP‐evoked CS bursting during S2 TPS was enhanced when S1 and S2 TPS trains were delivered in the presence of ISO (*P*_CSB_ = 0.65), although this effect was marginally significant (*p* = 0.049) (Figure 8E). Together, these results indicate that β‐AR activation enhances cooperative synaptic interactions during brief trains of TPS that are normally below threshold for LTP induction. Consistent with this, the potentiation of S2 synapses induced by S1 → S2 TPS in the presence of ISO (Figure 8E) was also significantly larger than that induced by 5 s of S2 TPS delivered in the presence of ISO without prior S1 TPS (Figure 3B) (*t*_(17)_ = 3.606, *p* = 0.00218).

**FIGURE 9:**
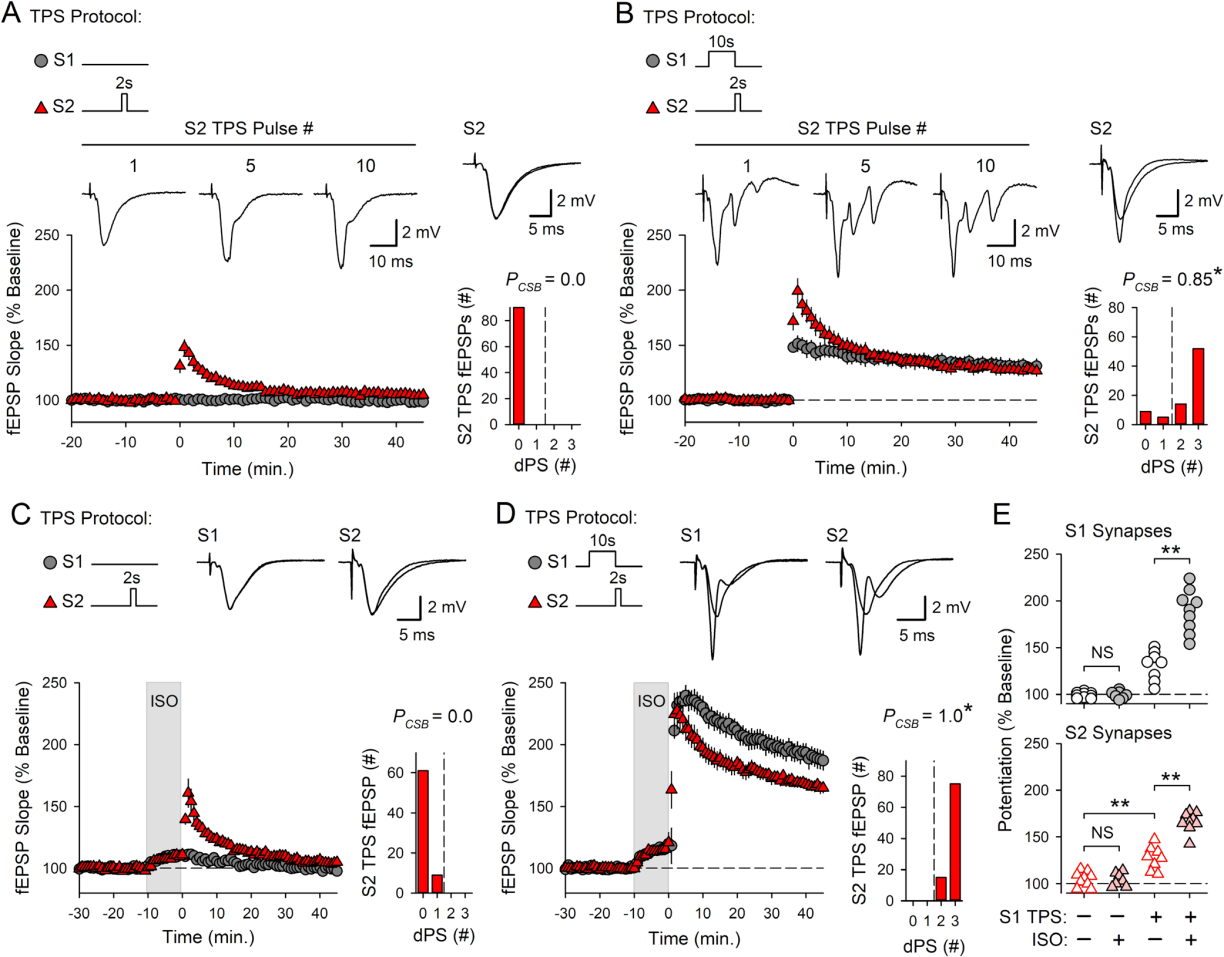
β‐AR activation and TPS interact in a highly synergistic fashion to promote LTP induction by cooperative synaptic interactions. (A) Control experiments (*n* = 9) where 2 s of TPS was delivered to S2 synapses (at time = 0). (B) 10 s of TPS was delivered to S1 synapses before 2 s of S2 TPS (*n* = 8). Traces in A and B show fEPSPs evoked by the 1st, 5th, and 10th stimulation pulses during S2 TPS and superimposed S2 fEPSPs evoked during baseline and 45 min after S2 TPS. (C) 2 s of TPS was delivered to S2 synapses at the end of a 10‐min bath application of ISO (*n* = 7). (D) 10 s of S1 TPS before 2 s of S2 TPS was delivered at the end of a 10‐min bath application of ISO (*n* = 9). Traces in C and D show superimposed fEPSPs elicited by S1 and S2 stimulation during baseline and 45 min post‐S2 TPS. Histograms in A–D show number of EPSPs evoking 0–3 dendritic PSs during S2 TPS. *p < 0.05 compared to control (A), one‐way ANOVA on Ranks, *H*_(3)_ = 28.182, *p* < 0.001. (E) S1 (top) and S2 (bottom) fEPSP slopes recorded 45 min after S2 TPS from all experiments. Results for S1 and S2 synapses were analyzed separately using two‐way ANOVAs followed by SNK comparisons (**p < 0.001, NS, not significant, *p* = 0.973 for S1 synapses; NS, *p* = 0.915 for S2 synapses). For S1 synapses there was a significant effect of S1 TPS (*F*_(1,29)_ = 144.244, *p* < 0.001), ISO (*F*_(1,29)_ = 33.750, *p* < 0.001), and a significant S1 TPS × ISO interaction (*F*_(1,29)_ = 33.188, *p* < 0.001). For S2 synapses there was a significant effect of S1 TPS (*F*_(1,29)_ = 149.566, *p* < 0.001), ISO (*F*_(1,29)_ = 30.693, *p* < 0.001), and a significant S1 TPS × ISO interaction (*F*_(1,29)_ = 32.442, *p* < 0.001).

As an additional test of the ability of β‐AR activation to enhance cooperativity in CS burst‐dependent LTP, I also examined whether ISO regulates cooperative synaptic interactions using a very brief train of S2 TPS (2 s) that, by itself, fails to induce LTP, even when delivered in the presence of ISO (Figure 9). In control experiments, 2 s of S2 TPS alone failed to elicit CS bursts (*P*_CSB_ = 0.0, *n* = 9) and had no lasting effect on synaptic strength (45 min post‐TPS S2 fEPSPs were 105% ± 3% of baseline) (Figure 9A). As expected, 10 s of S1 TPS delivered before S2 TPS facilitated EPSP‐evoked CS bursting during S2 TPS (*P*_CSB_ = 0.85, *n* = 8) and enabled the induction of a modest, but significant, potentiation of S2 synapses (S2 fEPSPs potentiated to 127% ± 4% of baseline, *p* < 0.001 compared to S2 TPS alone) (Figure 9B). In contrast, β‐AR activation did not enable LTP induction when 2 s of S2 TPS was delivered without prior S1 TPS (45 min post‐TPS S2 fEPSPs were 104% ± 3% of baseline, *n* = 7) (Figure 9C). β‐AR activation did, however, significantly enhance the heterosynaptic facilitation of LTP induction at S2 synapses induced by 10 s of S1 TPS (S2 fEPSPs potentiated to 166% ± 4% of baseline, *n* = 9) (Figure 9D,E). Together with the results shown in Figure 8, the highly synergistic effects of ISO and S1 TPS on LTP induction at S2 synapses indicate that β‐AR activation strongly enhances cooperative synaptic interactions during the induction of CS burst‐dependent LTP. Indeed, the combined effects of ISO and S1 TPS enable the induction of a robust and persistent potentiation at S2 synapses by just 10 EPSP‐evoked CS bursts (Figure 9D).

### 3.6 β‐AR Activation Prolongs the Time Course of Cooperative Synaptic Interactions During the Induction of CS Burst‐Dependent LTP

**FIGURE 10:**
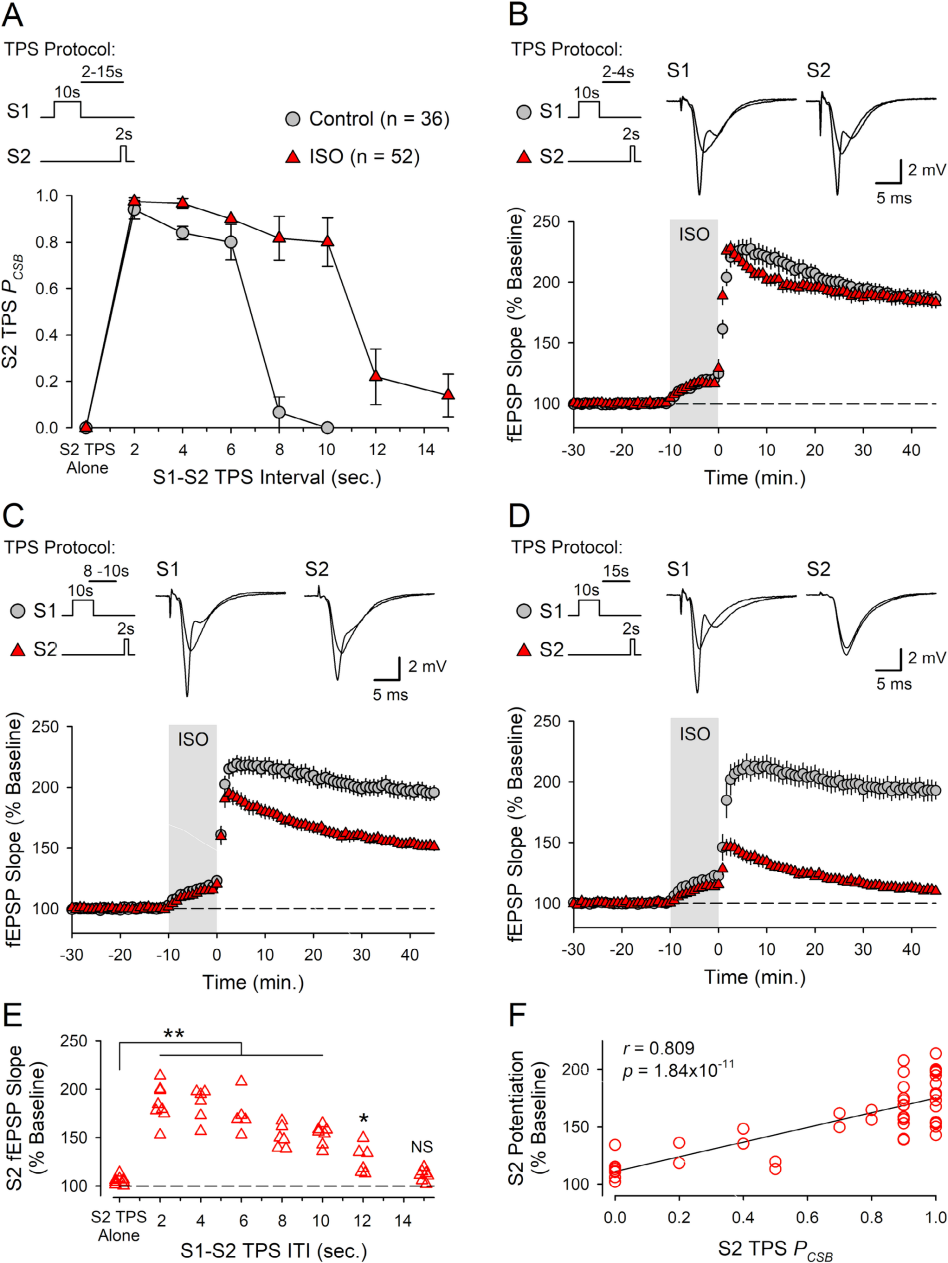
β‐AR activation prolongs the short‐term, heterosynaptic facilitation of EPSP‐evoked CS bursting induced by TPS and produces a broad time window for cooperative synaptic interactions during the induction CS burst‐dependent LTP. (A) Probability of CS bursting (*P*_CSB_) during S2 TPS trains delivered 2–15 s after S1 TPS in the presence and absence of ISO. In control experiments, EPSP‐evoked CS bursting during S2 TPS was significantly enhanced (*p* < 0.05, compared to S2 TPS alone results in Figure 9A) with S1 → S2 TPS ITIs up to 6 s (one‐way ANOVA on Ranks with Dunn's test comparisons to S2 TPS alone, *H*_(5)_ = 31.873, *p* < 0.001). In the presence of ISO, EPSP‐evoked CS bursting during S2 TPS was significantly enhanced with S1 → S2 ITIs up to 10 s (compared to S2 TPS alone in the presence of ISO, *n* = 9, *H*_(7)_ = 41.598, *p* < 0.001). (B) Combined results from experiments where 2 s of S2 TPS was delivered 2 (*n* = 8) or 4 s (*n* = 6) after a 10‐s‐long train of S1 TPS in the presence of ISO. (C) Same as in A but with S1 → S2 inter‐train intervals (ITIs) of 8 (*n* = 6) or 10 s (*n* = 7). (D) S2 TPS (2 s) was delivered 15 s after 10 s of S1 TPS (*n* = 7). Traces in B–D show superimposed fEPSPs elicited by S1 and S2 stimulation during baseline and 45 min post‐S2 TPS. (E) S2 fEPSP slopes recorded 45 min post‐S2 TPS from all experiments. Compared to control experiments (2 s of S2 TPS in the presence of ISO without prior S1 TPS, *n* = 7), S2 fEPSPs were significantly potentiated (**p < 0.001, *p = 0.041) with S1 → S2 TPS ITIs up to 12 s (one‐way ANOVA with SNK *post hoc* comparisons, *F*_(7,44)_ = 30.922, *p* < 0.001, NS, *p* = 0.545). (F) Scatter plot shows the percent increase in synaptic strength at S2 synapses plotted as a function of *P*_CSB_ during S2 TPS for all experiments where S1 TPS trains were delivered before S2 TPS.

β‐AR activation not only enhances the induction of conventional Hebbian LTP but also increases the time window during which synapses can interact in a cooperative/associative fashion and undergo LTP (Lin et al. 2003). Although the temporal constraints on the ability of synapses to interact in a cooperative fashion to induce conventional Hebbian LTP and CS burst‐dependent LTP are very different (tens of milliseconds vs. several seconds, respectively) (Lin et al. 2003; O'Dell 2022), this raises the interesting possibility that β‐AR activation also regulates the temporal properties of cooperativity in CS burst‐dependent LTP. Importantly, the heterosynaptic facilitation of EPSP‐evoked CS bursting induced by TPS underlies cooperative synaptic interactions during the induction of CS burst‐dependent LTP (O'Dell 2022). Thus, to determine whether β‐AR activation regulates the temporal properties of cooperativity in CS burst‐dependent LTP, I first examined the effects of ISO on the heterosynaptic facilitation of EPSP‐evoked CS bursting induced by TPS. In these experiments, 10 s of TPS was delivered to S1 synapses to induce EPSP‐evoked CS bursting, and 2 s of TPS was then delivered to S2 synapses with inter‐train intervals (ITIs) ranging from 2 to 15 s. In control experiments, EPSP‐evoked CS bursting during S2 TPS was strongly upregulated when S2 TPS was delivered 2–6 s after S1 TPS but was no longer significantly different from control (S2 TPS alone) when the S1‐S2 TPS ITI was increased to 8 s (Figure 10A). In contrast, when these same patterns of S1 and S2 TPS were delivered at the end of a 10‐min bath application of ISO, the heterosynaptic facilitation of EPSP‐evoked CS bursting during S2 TPS was still robust when the S1‐S2 TPS ITI was increased to 8 s and only began to fade when the ITI was increased to 12 s or more (Figure 10A). The heterosynaptic facilitation of LTP induction at S2 synapses induced by S1 TPS in the presence of ISO exhibited a similar time course, with S2 fEPSPs potentiating to 150% of baseline or more when S2 TPS was delivered 2–10 s after S1 TPS in the presence of ISO (Figure 10B,C). However, LTP induction at S2 synapses was reduced when the S1‐S2 TPS ITI was increased to 12 s and was no longer significant from control (S2 TPS alone in the presence of ISO) when the S1–S2 TPS ITI was increased to 15 s (Figure 10D,E). Consistent with the notion that LTP induction at S2 synapses is enabled by the heterosynaptic facilitation of EPSP‐evoked CS bursting produced by S1 TPS, there was a highly significant correlation between the magnitude of LTP at S2 synapses and the probability of EPSP‐evoked CS bursting during S2 TPS (Figure 10F). Thus, β‐AR activation not only prolongs the transient heterosynaptic facilitation of EPSP‐evoked CS bursting induced by TPS (Figure 10A) but also produces a broad temporal window lasting at least 10 s during which independent synapses can interact with one another in a cooperative fashion to undergo CS burst‐dependent LTP. Notably, the timescale of cooperativity in CS burst‐dependent LTP in the presence of ISO is nearly 2‐fold longer than that seen in control conditions (O'Dell 2022) and approximately 3 orders of magnitude longer than the timescale of cooperativity in conventional Hebbian LTP (Lin et al. 2003).

## 4 Discussion

**FIGURE 11:**
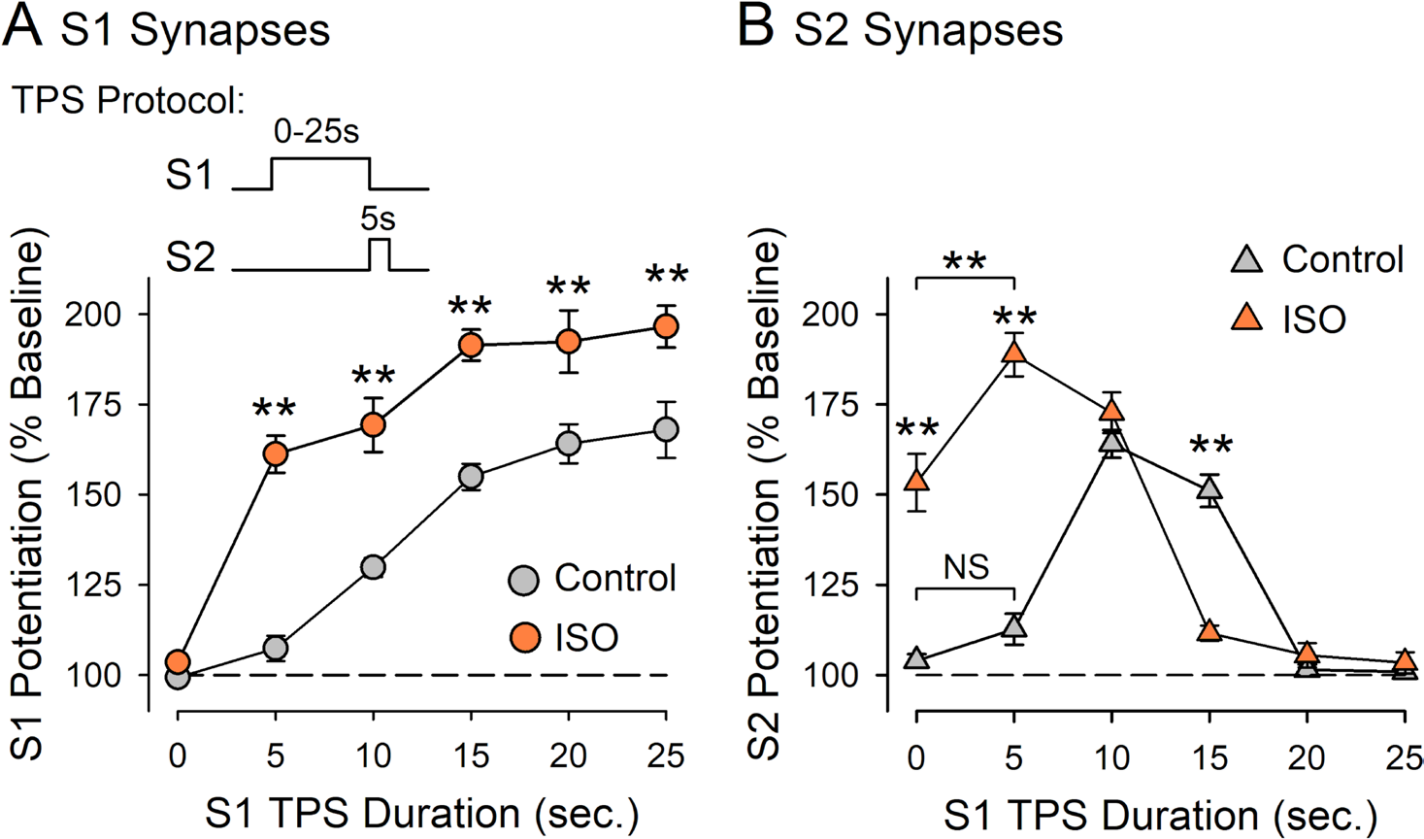
Effects of β‐AR activation on synaptic interactions during CS burst dependent‐LTP induction. Plots include results from experiments shown in Figures 2, 3, 6, 7C, and 8 and Figure S2 where different durations of S1 TPS were delivered before 5 s of S2 TPS. (A) Changes in strength at S1 synapses induced by 0–25 s of S1 TPS delivered either alone (control) or in the presence of 1.0 μM ISO. A two‐way ANOVA with SNK pairwise comparisons of control versus ISO (**p < 0.001) indicated a significant effect of S1 TPS duration (*F*_(5,129)_ = 89.383, *p* < 0.001), a significant effect of ISO (*F*_(1,129)_ = 132.786, *p* < 0.001), and a significant S1 TPS duration × ISO interaction (*F*_(5,129)_ = 6.195, *p* < 0.001). (B) Changes in S2 synaptic strength in the same experiments. A two‐way ANOVA followed by SNK post hoc comparisons revealed a significant effect of S1 duration (*F*_(5,129)_ = 73.289, *p* < 0.001), a significant effect of ISO (*F*_(1,129)_ = 48.677, *p* < 0.001), and a significant S1 TPS duration × ISO interaction (*F*_(5,129)_ = 54.36, *p* < 0.001). Five seconds of S1 TPS delivered before S2 TPS had no effect in control experiments (NS, *p* = 0.109) but facilitated LTP induction at S2 synapses when TPS was delivered in the presence of ISO (**p < 0.001). β‐AR activation inhibited LTP induction at S2 synapses when S2 TPS followed 15 s of S1 TPS (**p < 0.001). *N* = 79 control experiments and 62 experiments where TPS protocols were delivered in the presence of ISO.

In agreement with earlier studies (Thomas et al. 1996; Winder et al. 1999; Gelinas et al. 2008; Qian et al. 2012; Jami et al. 2023), I find that β‐AR activation enhances the induction of homosynaptic LTP by TPS protocols. However, the effects of ISO on synaptic interactions during different patterns of TPS indicate that β‐AR activation exerts equally robust modulatory effects on activity‐dependent forms of heterosynaptic plasticity that regulate CS burst‐dependent LTP induction. Interestingly, although β‐AR activation facilitated the induction of homosynaptic LTP at S1 synapses by trains of TPS lasting 5 or more seconds, it could enhance, suppress, or have no effect on the heterosynaptic facilitation of LTP induction induced by S1 TPS. The effects of β‐AR on synaptic interactions during CS burst‐dependent LTP induction are thus surprisingly complex. A reanalysis of the effects of ISO on changes in synaptic strength induced by different patterns of S1 → S2 TPS that include results from experiments using trains of S1 TPS lasting 20 and 25 s (Figure S2) provides a useful context for thinking about these curious results (Figure 11). In control experiments, the duration of TPS delivered to S1 synapses has markedly different effects on changes in synaptic strength at S1 and S2 synapses. At S1 synapses, LTP induction follows a simple rule—as the duration of S1 TPS is increased, the potentiation of S1 synapses grows (Figure 11A). In contrast, brief trains of S1 TPS that induce modest LTP at S1 synapses induce a strong heterosynaptic facilitation of LTP induction at S2 synapses while longer trains of S1 TPS that induce more robust homosynaptic potentiation at S1 synapses inhibit LTP induction at S2 synapses. Thus, LTP induction at S2 synapses exhibits a pronounced, inverted‐U shaped dependence on the duration of S1 TPS (Figure 11B). In the presence of ISO, LTP induction at S1 synapses is enhanced and, as in control experiments, longer duration trains of S1 TPS tend to induce larger increases in synaptic strength (Figure 11A). Notably, the inverted‐U shaped relationship between S1 TPS duration and S2 synapse potentiation is also preserved when TPS is delivered in the presence of ISO (Figure 11B). However, by enhancing both cooperative and competitive synaptic interactions, β‐AR activation produces a pronounced leftward shift in the inverted‐U shaped relationship between S1 TPS duration and S2 synapse potentiation (Figure 11B).

Interestingly, the analysis in Figure 11 indicates that while β‐AR activation enhances CS burst‐dependent LTP, it does so in a way that maintains the relationship between the magnitude of homosynaptic LTP and synaptic competition (i.e., synaptic competition is induced by patterns of synaptic stimulation that induce robust homosynaptic LTP). Why might this be important? Because TPS induces a Hebbian form of homosynaptic potentiation (Thomas et al. 1998; O'Dell 2022), CS burst‐dependent LTP likely shares a well‐characterized, but problematic, feature of standard Hebbian plasticity rules—runaway dynamics. Runaway changes in synaptic strength in neural networks with Hebbian synapses are generated by the correlational nature of Hebbian plasticity rules, that is, synapses potentiate in an unsupervised fashion in response to coincident pre‐ and postsynaptic activity. Thus, as some synapses undergo LTP, the increase in postsynaptic activity produced by these inputs increases the probability that other synapses undergo LTP. If left unopposed, this property of Hebbian LTP induction can trigger a self‐reinforcing, positive‐feedback cycle of potentiation that, as it continues, leads to runaway synaptic potentiation and loss of information encoded by differences in the strength of individual synapses (Miller and MacKay 1994; Turrigiano 2008; Zenke et al. 2013; Chen et al. 2013). Because of this, information storage in neural networks with Hebbian synapses requires rapid, negative‐feedback forms of homeostatic plasticity to oppose the runaway dynamics of Hebbian LTP (Chistiakova et al. 2015; Zenke and Gerstner 2017; Zenke et al. 2017). In CS burst‐dependent LTP, this negative feedback is likely provided, at least in part, by the strong heterosynaptic suppression of LTP induction that emerges during bouts of EPSP‐evoked CS bursting that induce robust homosynaptic LTP (O'Dell 2022). Thus, the leftward shift in the inverted‐U shaped relationship between S1 TPS duration and LTP induction at S2 synapses induced by ISO (Figure 11B) suggests that β‐AR activation not only enhances LTP induction but also recruits a compensatory increase in synaptic competition that can oppose the runaway dynamics of facilitated CS burst‐dependent LTP induction.

CS bursts not only provide the postsynaptic depolarization needed for NMDAR activation and LTP induction during TPS (Thomas et al. 1998; O'Dell 2022) but, as shown here, also trigger an A1 adenosine receptor‐mediated heterosynaptic depression that underlies synaptic competition (Figures 6 and 7). This dual role of CS bursts in LTP induction and synaptic competition thus provides a surprisingly simple, but computationally efficient, mechanism whereby increases in CS bursting triggered by β‐AR activation can facilitate LTP induction while simultaneously ensuring sufficient synaptic competition is available to oppose runaway synaptic dynamics. The shared dependence of TPS‐induced LTP and synaptic competition on postsynaptic CS bursts has other potentially important computational implications. For example, biologically realistic artificial neural networks with burst‐dependent synaptic plasticity rules can implement the error back‐propagation algorithm (Payeur et al. 2021; Sun et al. 2021), an especially powerful mathematical algorithm used in machine learning (Rumelhart et al. 1986). However, to successfully learn via error backpropagation, these networks require a novel form of synaptic competition regulated by the probability of postsynaptic bursting, rather than the firing rate of single action potentials (Payeur et al. 2021). Although the properties of synaptic competition in CS burst‐dependent LTP have not yet been fully characterized, the dual role of postsynaptic CS bursts in LTP induction and synaptic competition described here provides some initial experimental support for this theoretical prediction.

Many of the effects of β‐AR activation on TPS‐induced LTP are thought to be mediated by protein kinase A (PKA) and extracellular signal‐regulated kinase 1/2 signaling (O'Dell et al. 2015). However, a 10‐min bath application of ISO regulates several hundred phosphorylation sites in 100's of postsynaptic density proteins in the hippocampal CA1 region (Jami et al. 2023). Given this complexity, identification of the molecular mechanisms underlying the β‐AR modulation of synaptic interactions in CS burst‐dependent LTP will require further investigation. The amount of homosynaptic LTP induced by different patterns of TPS is, however, highly correlated with the probability of EPSP‐evoked CS bursting during TPS (O'Dell 2022), as is the magnitude of TPS‐induced heterosynaptic depression and synaptic competition (Figure 4C,D). Similarly, there is a significant correlation between the heterosynaptic facilitation of EPSP‐evoked CS bursting and cooperative LTP induction (Figure 10F). Together, these findings suggest that β‐AR‐mediated increases in CA1 pyramidal cell excitability may have a central role in regulating synaptic interactions during the induction of CS burst‐dependent LTP. Consistent with this notion, β‐AR activation elicits a PKA‐mediated inhibition of Kv4.2 and Kv1.1‐type potassium channels that enhances action potential backpropagation and dendritic spiking in CA1 pyramidal cells (Hoffman and Johnston 1999; Yuan et al. 2002; Liu et al. 2017). Additionally, because activation of voltage‐dependent calcium channels contributes to CS bursting in CA1 pyramidal cells (Takahashi and Magee 2009; Grienberger et al. 2014), PKA‐mediated increases in calcium channel activity induced by β‐AR activation (Patriarchi et al. 2016; Qian et al. 2017) may also have a role in facilitating cooperative and competitive synaptic interactions during TPS.

Consistent with the idea that an increase in pyramidal cell excitability underlies the multiple effects of β‐AR activation on CS burst‐dependent LTP, the GABA_A_ receptor antagonist gabazine not only enhances EPSP‐evoked CS bursting and homosynaptic LTP induction but, like ISO, also enhances TPS‐induced heterosynaptic depression (Figure 5) and synaptic competition (Figure 6). However, β‐AR activation had no effect on EPSP‐evoked CS bursting during 5 s of TPS but nonetheless enabled the induction of LTP during these brief trains of TPS (Figure 3A–C). This indicates that increases in pyramidal cell excitability cannot account for all the effects of β‐AR activation on CS burst‐dependent LTP. Thus, other mechanisms implicated in the β‐AR modulation of LTP induction, such as PKA‐mediated changes in AMPA receptor trafficking (Hu et al. 2007; Qian et al. 2012), increases in NMDAR activity (Raman et al. 1996; Murphy et al. 2014), and/or inhibition of protein phosphatase signaling (Thomas et al. 1996), likely also contribute to the effects of β‐AR activation on CS burst‐dependent LTP.

Although the ability of β‐AR activation to fundamentally alter the temporal and activity‐dependent properties of synaptic interactions in CS burst‐dependent plasticity is striking, there are several important questions to address in future experiments. First, bath‐applied receptor agonists and endogenously released transmitters can sometimes have very different effects on neuronal excitability and synaptic transmission (Rosen et al. 2015; Teixeira et al. 2018; Bacon et al. 2020). Although these findings raise concerns about the use of bath‐applied agonists to study the effects of modulatory neurotransmitters on synaptic plasticity, 10‐min‐long bath applications of NE mimic the β‐AR‐mediated enhancement of dendritic excitability (Liu et al. 2017) and the increase in EPSP‐spike coupling (Bacon et al. 2020) induced in CA1 pyramidal cells by optogenetic stimulation of LC fibers. ISO also mimics the effects of optogenetic activation of LC axons on LTP induction at cortical synapses (He et al. 2015). Thus, exogenous β‐AR agonists mimic the effects of endogenous NE on cellular processes likely to have an important role in CS burst‐dependent LTP. However, although bath application of ISO can mimic the persistent elevations in NE associated with tonic firing in LC neurons during heightened states of arousal and attention (Berridge and Waterhouse 2003; Feng et al. 2024), phasic bursts of activity in LC neurons also likely contribute to memory formation (Wilmot et al. 2024). Thus, the use of optogenetic activation of LC neurons will be important in future studies examining the noradrenergic regulation of CS burst‐dependent LTP. Second, the facilitation of both memory formation and LTP induction induced by optogenetic activation of LC fibers in the hippocampus may be mediated by the release of dopamine, rather than NE (Takeuchi et al. 2016; Wilmot et al. 2024; however, see Tsetsenis et al. 2022). Thus, a consideration of the role of dopamine receptor signaling will be important in future studies examining how modulatory inputs from the LC regulate CS burst‐dependent LTP. Finally, like BTSP, CS burst‐dependent LTP exhibits a retroactive form of synaptic cooperativity where CS bursts can trigger LTP induction at synapses that were active seconds earlier (O'Dell 2022). This form of cooperativity is thought to arise from the generation of synaptic eligibility traces, transient biochemical changes at synapses that, while having no direct effect on synaptic strength, persist for several seconds and enable synaptic potentiation in response to dendritic spikes induced by other synaptic inputs (Magee and Grienberger 2020). Notably, β‐AR activation can induce LTP in a retrograde fashion at cortical synapses by transforming eligibility traces generated by prior synaptic activity (He et al. 2015). Importantly, by providing a mechanism that allows temporally delayed signals related to prediction errors or reward to induce changes in synaptic strength, eligibility traces potentially solve the long‐standing “distal reward problem” in reinforcement learning (Magee and Grienberger 2020; Shouval and Kirkwood 2025). Thus, it will be interesting to determine whether a β‐AR‐mediated transformation of TPS‐induced eligibility traces also contributes to the surprisingly complex effects of β‐AR activation on the properties of CS burst‐dependent LTP.

## Author Contributions

T.J.O. conceived and designed research, performed experiments, analyzed data, interpreted results of experiments, prepared figures, wrote the manuscript, and approved the final version of the manuscript.

## Conflicts of Interest

The author declares no conflicts of interest.

## Supporting information


**Figure S1:** TPS‐induced LTP does not require β‐AR activation. (A) Hippocampal slices were continuously bathed in ACSF containing the β‐AR antagonist (S)‐(‐)‐propranolol (10 μM). TPS (30 s) was delivered to S1 synapses at time = 0.45 min post‐TPS S1 synapses were 158.6% ± 6% of baseline (*n* = 8). (B) Scatter plot shows results from all experiments where TPS was delivered in the presence and absence of propranolol (Pro). Control results (Con) are from the experiments shown in Figure 1D (*t*_(16)_ = 0.312, *p* = 0.759).

**Figure S2:** β‐AR activation enhances the induction of LTP at S1 synapses but has no effect on the suppression of LTP induction at S2 synapses induced by long trains of S1 TPS. (A) Control experiments where 20 s of TPS delivered to S1 synapses before 5 s of S2 TPS (at time = 0). 45 min post‐TPS S1 synapses potentiated to 164% ± 5% of baseline and S2 synapses were 101% ± 2% of baseline (*n* = 10). (B) 20 s of S1 TPS before S2 TPS was delivered at the end of a 10‐min bath application of 1.0 μM ISO (indicated by the shaded region). 45 min post‐TPS S1 were 192% ± 9% of baseline and S2 synapses were 105% ± 3% of baseline (*n* = 8). (C) S1 and S2 fEPSP slopes 45 min post‐S2 TPS from all experiments in *A* and *B*. Results were analyzed a using two‐way ANOVA with SNK *post hoc* comparisons (**p < 0.001, NS, not significant, *p* = 0.538). There was a significant difference between S1 and S2 synapses (*F*_(1,32)_ = 208.672, *p* < 0.001), a significant effect of ISO (*F*_(1,32)_ = 9.779, *p* = 0.004), and a significant synapse × ISO interaction (*F*_(1,32)_ = 5.490, *p* = 0.025). (D) Control experiments where 25 s of S1 TPS was delivered before 5 s of S2 TPS. 45 min post‐TPS S1 synapses were 168% ± 8% of baseline and S2 synapses were 101% ± 2% of baseline (*n* = 9). (E) 25 s of S1 TPS before S2 TPS was delivered in the presence of ISO. 45 min post‐TPS S1 synapses were 197% ± 6% of baseline and S2 synapses were 103% ± 3% of baseline (*n* = 8). (F) S1 and S2 fEPSP slopes 45 min post‐S2 TPS from all experiments in D and E. A two‐way ANOVA with SNK post hoc comparisons (**p < 0.001, NS, not significant, *p* = 0.738) revealed a significant difference between S1 and S2 synapses (*F*_(1,30)_ = 232.361, *p* < 0.001), a significant effect of ISO (*F*_(1,30)_ = 8.750, *p* = 0.006), and a significant synapse × ISO interaction (*F*_(1,30)_ = 6.152, *p* = 0.019). Histograms show number of EPSPs evoking 0–3 CS bursts during S2 TPS in all experiments. EPSP‐evoked CS bursting during S2 TPS was significantly reduced by ISO (20s S1 TPS: Mann Whitney *U* = 12.5, *p = 0.019; 25 s S1 TPS: Mann Whitney *U* = 11.5, *p = 0.017). This is likely due to the strong heterosynaptic depression of synaptic transmission at S2 synapses induced by 20 and 25 s of S1 TPS in the presence of ISO (fEPSPs evoked during S2 TPS were reduced to ~20% of baseline, data not shown).

**Video S1:** EPSP‐evoked complex spike bursting during a 30‐s‐long train of TPS.

## Data Availability

Data supporting the findings of this study are available upon reasonable request.

## References

[hipo70043-bib-0001] Babiec, W. E. , S. A. Jami , R. R. Guglietta , P. B. Chen , and T. J. O'Dell . 2017. “Differential Regulation of NMDA Receptor‐Mediated Transmission by SK Channels Underlies Dorsal‐Ventral Differences in Dynamics of Schaffer Collateral Synaptic Function.” Journal of Neuroscience 37: 1950–1964. 10.1523/JNEUROSCI.3196-16.2017. 28093473 PMC5320620

[hipo70043-bib-0002] Bacon, T. J. , A. E. Pickering , and J. R. Mellor . 2020. “Noradrenaline Release From Locus Coeruleus Terminals in the Hippocampus Enhances Excitation‐Spike Coupling in CA1 Pyramidal Neurons via Beta‐Adrenoceptors.” Cerebral Cortex 30: 6135–6151. 10.1093/cercor/bhaa159. 32607551 PMC7609922

[hipo70043-bib-0003] Berridge, C. W. , and B. D. Waterhouse . 2003. “The Locus Coeruleus‐Noradrenergic System: Modulation of Behavioral State and State‐Dependent Cognitive Processes.” Brain Research Reviews 42: 33–84. 10.1016/S0165-0173(03)00143-7. 12668290

[hipo70043-bib-0004] Bittner, K. C. , A. D. Milstein , C. Grienberger , S. Romani , and J. C. Magee . 2017. “Behavioral Time Scale Synaptic Plasticity Underlies CA1 Place Fields.” Science 357: 1033–1036. 10.1126/science.aan3846. 28883072 PMC7289271

[hipo70043-bib-0005] Bouret, S. , and S. J. Sara . 2004. “Reward Expectation, Orientation of Attention and Locus Coeruleus‐Medial Frontal Cortex Interplay During Learning.” European Journal of Neuroscience 20: 791–802. 10.1111/j.1460-9568.2004.03526.x. 15255989

[hipo70043-bib-0006] Brzosko, Z. , S. B. Mierau , and O. Paulsen . 2019. “Neuromodulation of Spike‐Timing‐Dependent Plasticity: Past, Present, and Future.” Neuron 103: 563–581. 10.1016/j.neuron.2019.05.041. 31437453

[hipo70043-bib-0007] Chen, J.‐Y. , P. Lonjers , C. Lee , M. Chistiakova , M. Volgushev , and M. Bazhenov . 2013. “Heterosynaptic Plasticity Prevents Runaway Synaptic Dynamics.” Journal of Neuroscience 33: 15915–15929. 10.1523/JNEUROSCI.5088-12.2013. 24089497 PMC3787503

[hipo70043-bib-0008] Chistiakova, M. , N. M. Bannon , J.‐Y. Chen , M. Bazhenov , and M. Volgushev . 2015. “Homeostatic Role of Heterosynaptic Plasticity: Models and Experiments.” Frontiers in Computational Neuroscience 9: 89. 10.3389/fncom.2015.00089. 26217218 PMC4500102

[hipo70043-bib-0009] Colgin, L. L. 2016. “Rhythms of the Hippocampal Network.” Nature Reviews Neuroscience 17: 239–249. 10.1038/nrn.2016.21. 26961163 PMC4890574

[hipo70043-bib-0010] Davis, C. H. , S. J. Starkey , M. F. Pozza , and G. L. Collingridge . 1991. “GABA Auto‐Receptors Regulate the Induction of LTP.” Nature 349: 609–611. 10.1038/349609a0. 1847993

[hipo70043-bib-0011] Dringenberg, H. C. 2020. “The History of Long‐Term Potentiation as a Memory Mechanism: Controversies, Confirmation, and Some Lessons to Remember.” Hippocampus 30: 987–1012. 10.1002/hipo.23213. 32442358

[hipo70043-bib-0012] Fanselow, M. S. , and K. M. Wassum . 2016. “The Origins and Organization of Vertebrate Pavlovian Conditioning.” Cold Spring Harbor Perspectives in Biology 8: a021717. https://cshperspectives.cshlp.org/content/8/1/a021717. 10.1101/cshperspect.a021717 PMC4691796 26552417

[hipo70043-bib-0013] Feng, J. , H. Don , J. E. Lischinsky , et al. 2024. “Monitoring Norepinephrine Release In Vivo Using Next‐Generation GRAB_NE_ Sensors.” Neuron 112: 1930–1942. 10.1016/j.neuron.2024.03.001. 38547869 PMC11364517

[hipo70043-bib-0014] Frémaux, N. , and W. Gerstner . 2016. “Neuromodulated Spike‐Timing‐Dependent Plasticity, and Theory of Three‐Factor Learning Rules.” Frontiers in Neural Circuits 9: 85. 10.3389/fncir.2015.00085. 26834568 PMC4717313

[hipo70043-bib-0015] Gallistel, C. R. , and L. D. Matzel . 2013. “The Neuroscience of Learning: Beyond the Hebbian Synapse.” Annual Review of Psychology 64: 169–200. 10.1146/annurev-psych-113011-143807. 22804775

[hipo70043-bib-0016] Gelinas, J. N. , G. Tenorio , N. Lemon , T. Abel , and P. V. Nguyen . 2008. “β‐Adrenergic Receptor Activation During Distinct Patterns of Stimulation Critically Modulates the PKA‐Dependence of LTP in the Mouse Hippocampus.” Learning & Memory 15: 281–289. 10.1101/lm.829208. 18441285 PMC2364601

[hipo70043-bib-0017] Gray, E. E. , and T. J. O'Dell . 2013. “Electrophysiological and Biochemical Studies of AMPA Receptor Phosphorylation and Synaptic Plasticity in Hippocampal CA1 Mini‐Slices.” In Multidisciplinary Tools for Investigating Synaptic Plasticity. Neuromethods, edited by P. Nguyen , vol. 81. Humana Press. 10.1007/978-1-62703-517-0_7.

[hipo70043-bib-0018] Grienberger, C. , X. Chen , and A. Konnerth . 2014. “NMDA Receptor‐Dependent Multidendrite Ca^2+^ Spikes Required for Hippocampal Burst Firing In‐Vivo.” Neuron 81: 1274–1281. 10.1016/j.neuron.2014.01.014. 24560703

[hipo70043-bib-0019] Grienberger, C. , and J. C. Magee . 2022. “Entorhinal Cortex Directs Learning‐Related Changes in CA1 Representations.” Nature 611: 554–562. 10.1038/s41586-022-05378-6. 36323779 PMC9668747

[hipo70043-bib-0020] Grover, L. M. , and T. J. Teyler . 1993. “Role of Adenosine in Heterosynaptic, Posttetanic Depression in Area CA1 of Hippocampus.” Neuroscience Letters 154: 39–42. 10.1016/0304-3940(93)90166-I. 8395668

[hipo70043-bib-0021] Gustafsson, B. , and H. Wigström . 1986. “Hippocampal Long‐Lasting Potentiation Produced by Pairing Single Volleys and Brief Conditioning Tetani Evoked in Separate Afferents.” Journal of Neuroscience 6: 1575–1582. 10.1523/JNEUROSCI.06-06-01575.1986. 3711996 PMC6568732

[hipo70043-bib-0022] Hagena, H. , N. Hansen , and D. Manahan‐Vaughan . 2016. “β‐Adrenergic Control of Hippocampal Function: Subserving the Choreography of Synaptic Information Storage and Memory.” Cerebral Cortex 26: 1349–1364. 10.1093/cercor/bhv330. 26804338 PMC4785955

[hipo70043-bib-0023] He, K. , M. Huertas , S. Z. Hong , et al. 2015. “Distinct Eligibility Traces for LTP and LTD in Cortical Synapses.” Neuron 88: 528–538. 10.1016/j.neuron.2015.09.037. 26593091 PMC4660261

[hipo70043-bib-0024] Hoffman, D. A. , and D. Johnston . 1999. “Neuromodulation of Dendritic Action Potentials.” Journal of Neurophysiology 81: 408–411. 10.1152/jn.1999.81.1.408. 9914302

[hipo70043-bib-0025] Hu, H. , E. Real , K. Takamiya , et al. 2007. “Emotion Enhances Learning via Norepinephrine Regulation of AMPA‐Receptor Trafficking.” Cell 131: 160–173. 10.1016/j.cell.2007.09.017. 17923095

[hipo70043-bib-0026] Jami, S. A. , B. J. Wilkinson , R. Guglietta , et al. 2023. “Functional and Phosphoproteomic Analysis of β‐Adrenergic Receptor Signaling at Excitatory Synapses in the CA1 Region of the Ventral Hippocampus.” Scientific Reports 13: 7493. 10.1038/s41598-023-34401-7. 37161045 PMC10170123

[hipo70043-bib-0027] Jorden, R. 2024. “The Locus Coeruleus as a Global Model Failure System.” Trends in Neurosciences 47: 92–105. 10.1016/j.tins.2023.11.006. 38102059

[hipo70043-bib-0028] Kaufman, A. M. , T. Geiller , and A. Losonczy . 2022. “A Role for the Locus Coeruleus in Hippocampal CA1 Place Cell Reorganization During Spatial Reward Learning.” Neuron 105: 1018–1026. 10.1016/j.neuron.2019.12.029. PMC7265133 31980319

[hipo70043-bib-0029] Larson, J. , and E. Munkácsy . 2015. “Theta‐Burst LTP.” Brain Research 1621: 38–50. 10.1016/j.brainres.2014.10.034. 25452022 PMC4411212

[hipo70043-bib-0030] Lin, Y.‐W. , M.‐Y. Min , T.‐H. Chiu , and H.‐W. Yang . 2003. “Enhancement of Associative Long‐Term Potentiation by Activation of β‐Adrenergic Receptors at CA1 Synapses in Rat Hippocampal Slices.” Journal of Neuroscience 23: 4173–4181. 10.1523/JNEUROSCI.23-10-04173.2003. 12764105 PMC6741099

[hipo70043-bib-0031] Liu, Y. , L. Cui , M. K. Schwarz , Y. Don , and O. M. Schlüter . 2017. “Adrenergic Gate Release for Spike Timing‐Dependent Synaptic Potentiation.” Neuron 93: 394–408. 10.1016/j.neuron.2016.12.039. 28103480 PMC5267933

[hipo70043-bib-0032] Lovatt, D. , Q. Xu , W. Liu , et al. 2012. “Neuronal Adenosine Release, and Not Astrocytic ATP Release, Mediates Feedback Inhibition of Excitatory Activity.” Proceedings of the National Academy of Sciences 109: 6265–6270. 10.1073/pnas.1120997109. PMC3341061 22421436

[hipo70043-bib-0033] Lovett‐Barron, M. , G. F. Turi , P. Kaifosh , et al. 2012. “Regulation of Neuronal Input Transformation by Tunable Dendritic Inhibition.” Nature Neuroscience 15: 423–430. 10.1038/nn.3024. 22246433

[hipo70043-bib-0034] Madar, A. D. , A. Jiang , C. Dong , and M. E. J. Sheffield . 2025. “Synaptic Plasticity Rules Driving Representational Shifting in the Hippocampus.” Nature Neuroscience 28: 848–860. 10.1038/s41593-025-01894-6. 40113934

[hipo70043-bib-0035] Magee, J. C. , and C. Grienberger . 2020. “Synaptic Plasticity Forms and Functions.” Annual Review of Neuroscience 43: 95–117. 10.1146/annurev-neuro-090919-022842. 32075520

[hipo70043-bib-0036] McCarren, M. , and B. E. Alger . 1985. “Use‐Dependent Depression of IPSPs in Rat Hippocampal Pyramidal Cells In Vitro.” Journal of Neurophysiology 53: 557–571. 10.1152/jn.1985.53.2.557. 2984352

[hipo70043-bib-0037] Miller, K. D. , and D. J. C. MacKay . 1994. “The Role of Constraints in Hebbian Learning.” Neural Computation 6: 100–126. 10.1162/neco.1994.6.1.100.

[hipo70043-bib-0038] Mitchell, J. B. , C. R. Lupica , and T. V. Dunwiddie . 1993. “Activity‐Dependent Release of Endogenous Adenosine Modulates Synaptic Responses in the Rat Hippocampus.” Journal of Neuroscience 13: 3439–3447. 10.1523/JNEUROSCI.13-08-03439. 8393482 PMC6576537

[hipo70043-bib-0039] Mott, D. D. , and D. V. Lewis . 1991. “Facilitation of the Induction of Long‐Term Potentiation by GABA_B_ Receptors.” Science 252: 1718–1720. 10.1126/science.1675489. 1675489

[hipo70043-bib-0040] Murphy, J. A. , I. S. Stein , C. G. Lau , et al. 2014. “Phosphorylation of Ser1166 on GluN2B by PKA Is Critical to Synaptic NDMA Receptor Function and Ca^2+^ Signaling in Spines.” Journal of Neuroscience 34: 869–879. 10.1523/JNEUROSCI.4538-13.2014. 24431445 PMC3891964

[hipo70043-bib-0041] O'Dell, T. J. 2022. “Behavioral Timescale Cooperativity and Competitive Synaptic Interactions Regulate the Induction of Complex Spike Burst‐Dependent Long‐Term Potentiation.” Journal of Neuroscience 42: 2647–2661. 10.1523/JNEUROSCI.1950-21.2022. 35135856 PMC8973416

[hipo70043-bib-0042] O'Dell, T. J. , S. A. Connor , R. Guglietta , and P. V. Nguyen . 2015. “β‐Adrenergic Receptor Signaling and Modulation of Long‐Term Potentiation in the Mammalian Hippocampus.” Learning & Memory 22: 461–471. 10.1101/lm.031088.113. 26286656 PMC4561407

[hipo70043-bib-0043] Otto, T. , H. Eichenbaum , S. I. Wiener , and C. G. Wible . 1991. “Learning‐Related Patterns of CA1 Spike Trains Parallel Stimulation Parameters Optimal for Inducing Hippocampal Long‐Term Potentiation.” Hippocampus 1: 181–192. 10.1002/hipo.450010206. 1669292

[hipo70043-bib-0044] Patriarchi, T. , H. Qian , V. Di Biase , et al. 2016. “Phosphorylation of Cav1. 2 on S1928 Uncouples the L‐Type Ca^2+^ Channel From the β2 Adrenergic Receptor.” EMBO Journal 35: 1330–1345. 10.15252/embj.201593409. 27103070 PMC4910527

[hipo70043-bib-0045] Payeur, A. , J. Guerguiev , F. Zenke , B. A. Richards , and R. Naud . 2021. “Burst‐Dependent Synaptic Plasticity Can Coordinate Learning in Hierarchical Circuits.” Nature Neuroscience 24: 1010–1019. 10.1038/s41593-021-00970-x. 33986551

[hipo70043-bib-0046] Priestley, J. B. , J. C. Bowler , S. V. Rolotti , S. Fusi , and A. Losonczy . 2022. “Signatures of Rapid Plasticity in Hippocampal CA1 Representations During Novel Experiences.” Neuron 110: 1978–1992. 10.1016/j.neuron.2022.03.026. 35447088 PMC9233041

[hipo70043-bib-0047] Qian, H. , L. Matt , M. Zhang , et al. 2012. “β2‐Adrenergic Receptor Supports Prolonged Theta Tetanus‐Induced LTP.” Journal of Neurophysiology 107: 2703–2712. 10.1152/jn.00374.2011. 22338020 PMC3362273

[hipo70043-bib-0048] Qian, H. , T. Patriarchi , J. L. Prince , et al. 2017. “Phosphorylation of Ser1928 Mediates the Enhanced Activity of the L‐Type Ca^2+^ Channel Cav1.2 by the β2‐Adrenergic Receptor in Neurons.” Science Signaling 10: eaaf9659. 10.1126/scisignal.aaf9659. 28119465 PMC5310946

[hipo70043-bib-0049] Raman, I. M. , G. Tong , and C. E. Jahr . 1996. “β‐Adrenergic Regulation of Synaptic NMDA Receptors by cAMP‐Dependent Protein Kinase.” Neuron 16: 415–421. 10.1016/S0896-6273(00)80059-8. 8789956

[hipo70043-bib-0050] Rosen, Z. B. , S. Cheung , and S. A. Siegelbaum . 2015. “Midbrain Dopamine Neuros Bidirectionally Regulate CA3‐CA1 Synaptic Drive.” Nature Neuroscience 18: 1763–1771. 10.1038/nn.4152. 26523642 PMC11186581

[hipo70043-bib-0051] Rumelhart, D. E. , G. E. Hinton , and R. J. Williams . 1986. “Learning Representations by Back‐Propagating Errors.” Nature 323: 533–536. 10.1038/323533a0.

[hipo70043-bib-0052] Sara, S. J. 2009. “The Locus Coeruleus and Noradrenergic Modulation of Cognition.” Nature Reviews Neuroscience 10: 211–223. 10.1038/nrn2573. 19190638

[hipo70043-bib-0053] Sara, S. J. , and M. Segal . 1991. “Chapter 40 – Plasticity of Sensory Responses of Locus Coeruleus Neurons in the Behaving Rat: Implications for Cognition.” Progress in Brain Research 88: 571–585. 10.1016/S0079-6123(08)63835-2. 1813935

[hipo70043-bib-0054] Sara, S. J. , A. Vankov , and A. Hervé . 1994. “Locus Coeruleus‐Evoked Responses in Behaving Rats: A Clue to the Role of Noradrenaline in Memory.” Brain Research Bulletin 35: 457–464. 10.1016/0361-9230(94)90159-7. 7859103

[hipo70043-bib-0055] Shouval, H. Z. , and A. Kirkwood . 2025. “Eligibility Traces as a Synaptic Substrate for Learning.” Current Opinion in Neurobiology 91: 102978. 10.1016/j.conb.2025.102978. 39965463

[hipo70043-bib-0056] Sun, W. , X. Zhao , and N. Spruston . 2021. “Bursting Potentiates the Neuro‐AI Connection.” Nature Neuroscience 24: 905–906. 10.1038/s41593-021-00844-2. 33986550

[hipo70043-bib-0057] Takahashi, H. , and J. C. Magee . 2009. “Pathway Interactions and Synaptic Plasticity in the Dendritic Tuft Regions of CA1 Pyramidal Neurons.” Neuron 62: 102–111. 10.1016/j.neuron.2009.03.007. 19376070

[hipo70043-bib-0058] Takeuchi, T. , A. J. Duszkiewicz , A. Sonneborn , et al. 2016. “Locus Coeruleus and Dopaminergic Consolidation of Everyday Memory.” Nature 37: 357–362. 10.1038/nature19325. PMC5161591 27602521

[hipo70043-bib-0059] Tanaka, K. Z. , H. He , A. Tomar , K. Niisato , A. J. Y. Huang , and T. J. McHugh . 2018. “The Hippocampal Engram Maps Experience but Not Place.” Science 361: 392–397. 10.1126/science.aat5397. 30049878

[hipo70043-bib-0060] Teixeira, C. M. , Z. B. Rosen , D. Suri , et al. 2018. “Hippocampal 5‐HT Input Regulates Memory Formation and Schaffer Collateral Excitation.” Neuron 98: 992–1004. 10.1016/j.neuron.2018.04.030. 29754752 PMC6383566

[hipo70043-bib-0061] Thomas, M. J. , T. D. Moody , M. Makhinson , and T. J. O'Dell . 1996. “Activity‐Dependent β‐Adrenergic Modulation of Low‐Frequency Stimulation Induced LTP in the Hippocampal CA1 Region.” Neuron 17: 475–482. 10.1016/S0896-6273(00)80179-8. 8816710

[hipo70043-bib-0062] Thomas, M. J. , A. M. Watabe , T. D. Moody , M. Makhinson , and T. J. O'Dell . 1998. “Postsynaptic Complex Spike Bursting Enables the Induction of LTP by Theta Frequency Synaptic Stimulation.” Journal of Neuroscience 18: 7118–7126. 10.1523/JNEUROSCI.18-18-07118.1998. 9736635 PMC6793261

[hipo70043-bib-0063] Tsetsenis, T. , J. K. Badyna , R. Li , and J. A. Dani . 2022. “Activation of a Locus Coeruleus to Dorsal Hippocampus Noradrenergic Circuit Facilitates Associative Learning.” Frontiers in Cellular Neuroscience 16: 887679. 10.3389/fncel.2022.887679. 35496910 PMC9051520

[hipo70043-bib-0064] Turrigiano, G. G. 2008. “The Self‐Tuning Neuron: Synaptic Scaling of Excitatory Synapses.” Cell 135: 422–435. 10.1016/j.cell.2008.10.008. 18984155 PMC2834419

[hipo70043-bib-0065] Wigström, H. , and B. Gustafsson . 1984. “Facilitated Induction of Hippocampal Long‐Lasting Potentiation During Blockade of Inhibition.” Nature 301: 603–604. 10.1038/301603a0. 6298626

[hipo70043-bib-0066] Wilmot, J. H. , C. R. A. F. Diniz , A. P. Crestani , et al. 2024. “Phasic Locus Coeruleus Activity Enhances Trace Fear Conditioning by Increasing Dopamine Release in the Hippocampus.” eLife 12: RP91465. 10.7554/eLife.91465. 38592773 PMC11003744

[hipo70043-bib-0067] Winder, D. G. , K. C. Martin , I. A. Muzio , et al. 1999. “ERK Plays a Regulatory Role in Induction of LTP by Theta Frequency Stimulation and Its Modulation by β‐Adrenergic Receptors.” Neuron 24: 715–726. 10.1016/S0896-6273(00)81124-1. 10595521

[hipo70043-bib-0068] Wu, L.‐G. , and P. Saggau . 1994. “Adenosine Inhibits Evoked Synaptic Transmission Primarily by Reducing Presynaptic Calcium Influx in Area CA1 of Hippocampus.” Neuron 12: 1139–1148. 10.1016/0896-6273(94)90321-2. 8185949

[hipo70043-bib-0069] Wu, Z. , Y. Cui , H. Wang , et al. 2023. “Neuronal Activity‐Induced, Equilibrative Nucleoside Transporter‐Dependent, Somatodendritic Adenosine Release Revealed by a GRAB Sensor.” Proceedings of the National Academy of Sciences 120: e2212387120. 10.1073/pnas.2212387120. PMC10083574 36996110

[hipo70043-bib-0070] Xiao, K. , Y. Li , R. A. Chitwood , and J. C. Magee . 2023. “A Critical Role for CaMKII in Behavioral Timescale Synaptic Plasticity in Hippocampal CA1 Pyramidal Cells.” Science Advances 9: eadi3088. 10.1126/sciadv.adi3088. 37672577 PMC10482326

[hipo70043-bib-0071] Yuan, L.‐L. , J. P. Adams , M. Swank , J. D. Sweatt , and D. Johnston . 2002. “Protein Kinase Modulation of Dendritic K^+^ Channels in Hippocampus Involves a Mitogen‐Activated Protein Kinase Pathway.” Journal of Neuroscience 22: 4860–4868. 10.1523/JNEUROSCI.22-12-04860.2002. 12077183 PMC6757742

[hipo70043-bib-0072] Zenke, F. , and W. Gerstner . 2017. “Hebbian Plasticity Requires Compensatory Processes on Multiple Timescales.” Philosophical Transactions of the Royal Society, B: Biological Sciences 372: 20160259. 10.1098/rstb.2016.0259. PMC5247595 28093557

[hipo70043-bib-0073] Zenke, F. , W. Gerstner , and S. Ganguli . 2017. “The Temporal Paradox of Hebbian Learning and Homeostatic Plasticity.” Current Opinion in Neurobiology 43: 166–176. 10.1016/j.conb.2017.03.015. 28431369

[hipo70043-bib-0074] Zenke, F. , G. Hennequin , and W. Gerstner . 2013. “Synaptic Plasticity in Neural Networks Needs Homeostasis With a Fast Rate Detector.” PLoS Computational Biology 9, no. 11: e1003330. 10.1371/journal.pcbi.1003330. 24244138 PMC3828150

[hipo70043-bib-0075] Zhao, X. , C.‐L. Hsu , and N. Spruston . 2022. “Rapid Synaptic Plasticity Contributes to a Learned Conjunctive Code of Position and Choice‐Related Information in the Hippocampus.” Neuron 110: 96–108. 10.1016/j.neuron.2021.10.003. 34678146

