# Supplementary figures and images for: β‐Adrenergic Receptor Activation Modulates the Induction of Complex Spike‐Dependent LTP by Regulating Multiple Forms of Heterosynaptic Plasticity
Source: Hippocampus. 2025 Oct 15;35(6):e70043. doi: 10.1002/hipo.70043 (PMC12522536; doi:10.1002/hipo.70043)

## A Propranolol

TPS Protocol:

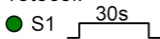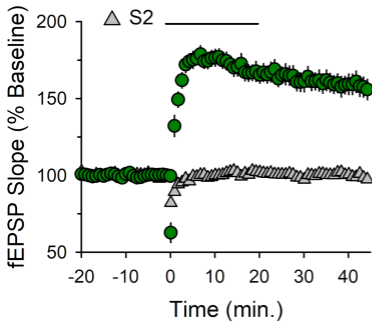

## B S2 Potentiation

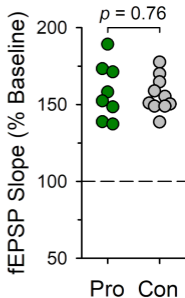

Supplement: Supplementary file 1 — Figure S1: TPS‐induced LTP does not require β‐AR activation. (A) Hippocampal slices were continuously bathed in ACSF containing the β‐AR antagonist (S)‐(‐)‐propranolol (10 μM). TPS (30 s) was delivered to S1 synapses at time = 0.45 min post‐TPS S1 synapses were 158.6% ± 6% of baseline (n = 8). (B) Scatter plot shows results from all experiments where TPS was delivered in the presence and absence of propranolol (Pro). Control results (Con) are from the experiments shown in Figure 1D (t (16) = 0.312, p = 0.759). [file HIPO-35-0-s001.pdf]

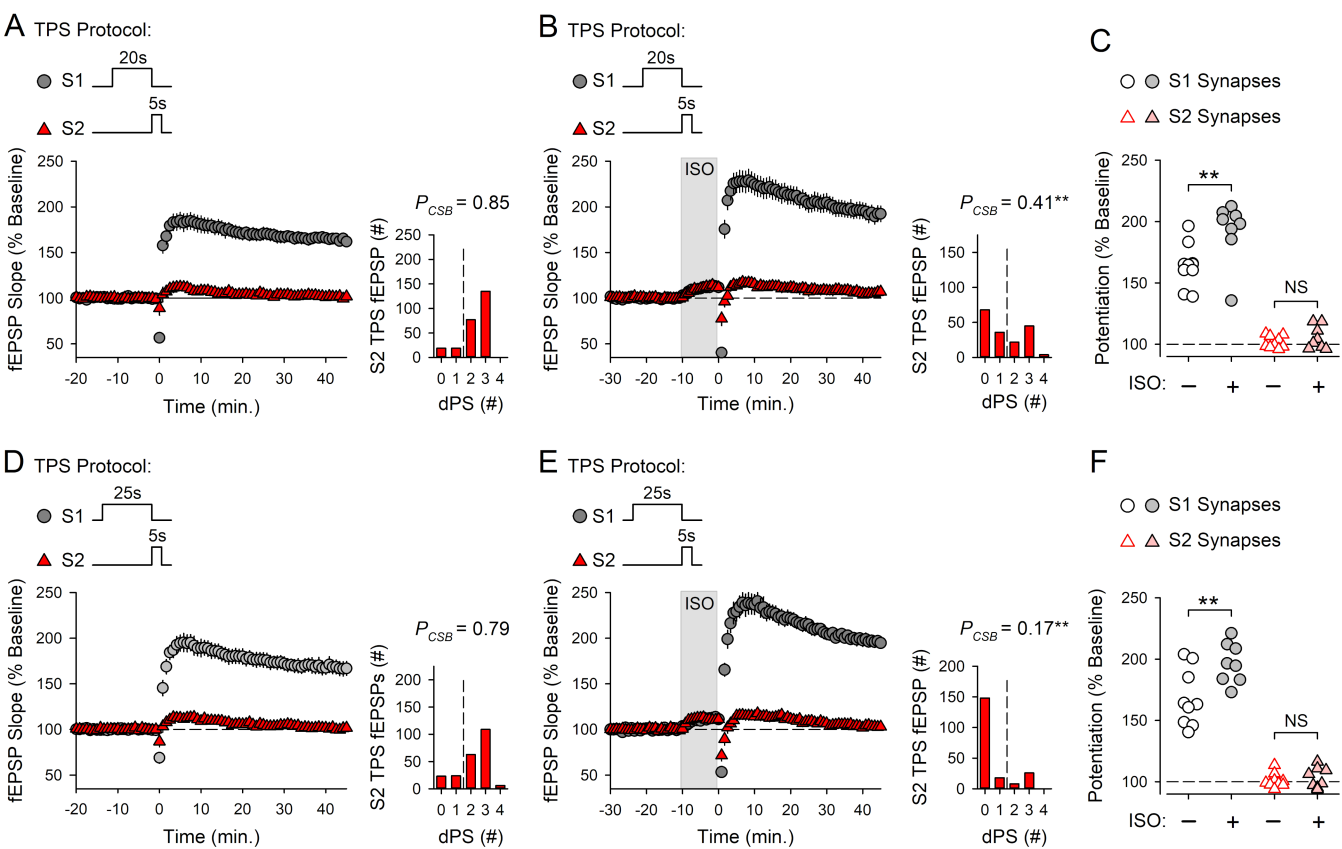

Supplement: Supplementary file 2 — Figure S2: β‐AR activation enhances the induction of LTP at S1 synapses but has no effect on the suppression of LTP induction at S2 synapses induced by long trains of S1 TPS. (A) Control experiments where 20 s of TPS delivered to S1 synapses before 5 s of S2 TPS (at time = 0). 45 min post‐TPS S1 synapses potentiated to 164% ± 5% of baseline and S2 synapses were 101% ± 2% of baseline (n = 10). (B) 20 s of S1 TPS before S2 TPS was delivered at the end of a 10‐min bath application of 1.0 μM ISO (indicated by the shaded region). 45 min post‐TPS S1 were 192% ± 9% of baseline and S2 synapses were 105% ± 3% of baseline (n = 8). (C) S1 and S2 fEPSP slopes 45 min post‐S2 TPS from all experiments in A and B. Results were analyzed a using two‐way ANOVA with SNK post hoc comparisons (**p < 0.001, NS, not significant, p = 0.538). There was a significant difference between S1 and S2 synapses (F (1,32) = 208.672, p < 0.001), a significant effect of ISO (F (1,32) = 9.779, p = 0.004), and a significant synapse × ISO interaction (F (1,32) = 5.490, p = 0.025). (D) Control experiments where 25 s of S1 TPS was delivered before 5 s of S2 TPS. 45 min post‐TPS S1 synapses were 168% ± 8% of baseline and S2 synapses were 101% ± 2% of baseline (n = 9). (E) 25 s of S1 TPS before S2 TPS was delivered in the presence of ISO. 45 min post‐TPS S1 synapses were 197% ± 6% of baseline and S2 synapses were 103% ± 3% of baseline (n = 8). (F) S1 and S2 fEPSP slopes 45 min post‐S2 TPS from all experiments in D and E. A two‐way ANOVA with SNK post hoc comparisons (**p < 0.001, NS, not significant, p = 0.738) revealed a significant difference between S1 and S2 synapses (F (1,30) = 232.361, p < 0.001), a significant effect of ISO (F (1,30) = 8.750, p = 0.006), and a significant synapse × ISO interaction (F (1,30) = 6.152, p = 0.019). Histograms show number of EPSPs evoking 0–3 CS bursts during S2 TPS in all experiments. EPSP‐evoked CS bursting during S2 TPS was significantly reduced by ISO (20s S1 TPS: Mann [file HIPO-35-0-s002.pdf]
